# Phytoagents for Cancer Management: Regulation of Nucleic Acid Oxidation, ROS, and Related Mechanisms

**DOI:** 10.1155/2013/925804

**Published:** 2013-12-25

**Authors:** Wai-Leng Lee, Jing-Ying Huang, Lie-Fen Shyur

**Affiliations:** ^1^School of Science, Monash University Sunway Campus, Jalan Lagoon Selatan, 47500 Bandar Sunway, Selangor Darul Ehsan, Malaysia; ^2^Agricultural Biotechnology Research Center, Academia Sinica, No. 128, Sec. 2, Academia Road, Nankang, Taipei 115, Taiwan; ^3^Graduate Institute of Pharmacognosy, Taipei Medical University, No. 250 Wu-Hsing Street, Taipei 110, Taiwan; ^4^Graduate Institute of Biotechnology, National Chung Hsing University, No. 250 Kuo Kuang Rd., Taichung 402, Taiwan

## Abstract

Accumulation of oxidized nucleic acids causes genomic instability leading to senescence, apoptosis, and tumorigenesis. Phytoagents are known to reduce the risk of cancer development; whether such effects are through regulating the extent of nucleic acid oxidation remains unclear. Here, we outlined the role of reactive oxygen species in nucleic acid oxidation as a driving force in cancer progression. The consequential relationship between genome instability and cancer progression highlights the importance of modulation of cellular redox level in cancer management. Current epidemiological and experimental evidence demonstrate the effects and modes of action of phytoagents in nucleic acid oxidation and provide rationales for the use of phytoagents as chemopreventive or therapeutic agents. Vitamins and various phytoagents antagonize carcinogen-triggered oxidative stress by scavenging free radicals and/or activating endogenous defence systems such as Nrf2-regulated antioxidant genes or pathways. Moreover, metal ion chelation by phytoagents helps to attenuate oxidative DNA damage caused by transition metal ions. Besides, the prooxidant effects of some phytoagents pose selective cytotoxicity on cancer cells and shed light on a new strategy of cancer therapy. The “double-edged sword” role of phytoagents as redox regulators in nucleic acid oxidation and their possible roles in cancer prevention or therapy are discussed in this review.

## 1. Nucleic Acid Oxidation as a Marker of Oxidative Insult by Reactive Oxygen Species and the Driving Force in Cancer Progression

**Figure 1:**
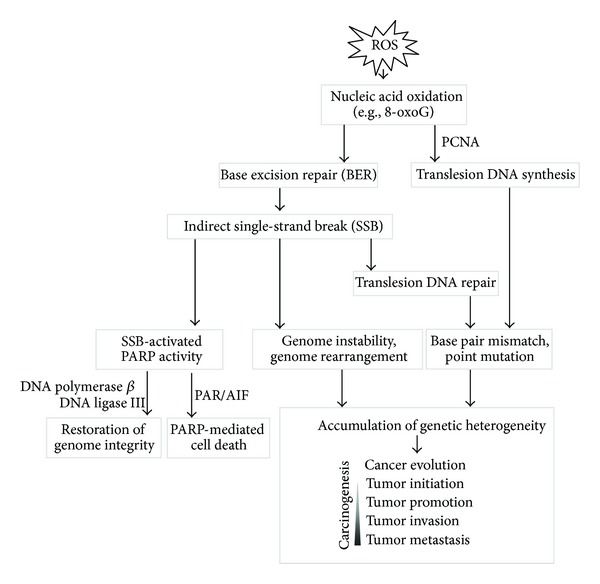
Genetic heterogeneity following nucleic acid oxidation is a major driving force of cancer progression. ROS causes the oxidation of DNA bases. Subsequent base excision repair (BER) introduces genetic errors during the repair process, and the accumulation of these errors drives cancer progression.

The integrity of the genome is of crucial importance for proper gene expression and DNA replication. Loss of genome integrity jeopardizes normal cellular physiological activities and leads to cellular pathological events such as senescence, apoptosis, and tumorigenesis [1]. Under oxidative stress, the level of genotoxic reactive oxygen species (ROS) is abnormally elevated. ROS interact with and modify the chemical properties of biomolecules inside the cell, which causes oxidative insults such as oxidation of nucleic acids, peroxidation of lipids [2], and denaturation of proteins [3]. Oxidative modification to DNA structure mainly occurs in the form of base oxidation. Guanine, which possesses the lowest oxidation potential of the DNA bases, is the most frequent target of ROS. ROS-elicited changes in biomolecules can be used as biomarkers to indicate the presence and extent of oxidative insult. 8-Oxo-7,8-dihydroguanine (8-oxoG), the oxidation product of the DNA base guanine is a well-characterized marker for oxidative stress-induced DNA damage [4]. Following the oxidation of a DNA base, genome integrity is at increased risk because the DNA repair process, base excision repair (BER), can increase the level of interrupted DNA strands resulting in indirect single-strand break (SSB) [5], subsequently leading to introduction of mismatched base pairing during translesion DNA repair [6]. As a consequence, genome instability and accumulation of mutations lead to genetic heterogeneity in cancer cells that drive the adaptive evolution of cancer colonies with survival/expansion advantages [7].  Figure 1 shows the genetic instability and heterogeneity caused by nucleic acid oxidation in cancer cells which lead to carcinogenesis and cancer evolution. During BER, indirect SSB are produced as intermediates after the removal of oxidized bases and their corresponding nucleotides. If SSB takes place at adjacent regions on both strands of the same chromosome, genome instability can ensue. Meanwhile, poly (ADP-ribose) polymerase (PARP) is activated after binding to SSB and consumes NAD+ to synthesize polyA chains which then recruit important DNA repair enzymes, such as DNA polymerase *β* and DNA ligase III. PARP also induces apoptosis through increased poly (ADP-ribose) (PAR) levels that facilitate the release of apoptosis-inducing factor (AIF) from mitochondria and elicit apoptosis. Otherwise, depletion of NAD due to excessive PARP activity will further deplete the ATP pool and lead to cell lysis (necrosis). Proliferating cell nuclear antigen (PCNA) promotes the switch to a specialized DNA polymerase with a larger active site that tolerates damaged bases at the expense of sacrificing fidelity during translesion synthesis/repair. Lower fidelity increases the chance of mismatch which gives rise to point mutations. The accumulation of genome instability and point mutations results in genome heterogeneity among cells and, chronologically, within cells. Tumor initiation is triggered by mutations that can activate oncogenes or silence tumor suppressor genes. Further mutations that give rise to gain/loss of function of genes then grant tumor cells the ability to resist growth control. Further gain/loss of function continues to drive cancer progression enabling tumor cells to escape layers of control and become capable of invasion and metastasis.

Elevated levels of oxidative DNA lesions (8-oxoG) have been noted in various tumors, supporting the argument that such damage contributes to the etiology of cancer. Therefore, 8-oxoG has been established as an important biomarker which is widely used to measure oxidative stress and assess risk of tumor initiation after exposure to various carcinogenic substances and pollutants [8]. In a cohort study involving esophageal cancer patients, more extensive oxidative damage to DNA as indicated by 8-oxoG levels was detected in cancer patients, in comparison to a healthy control group. Smoking habits and alcohol consumption, risk factors for esophageal cancer, were also correlated with the observed levels of oxidative DNA damage [9].

**Figure 2:**
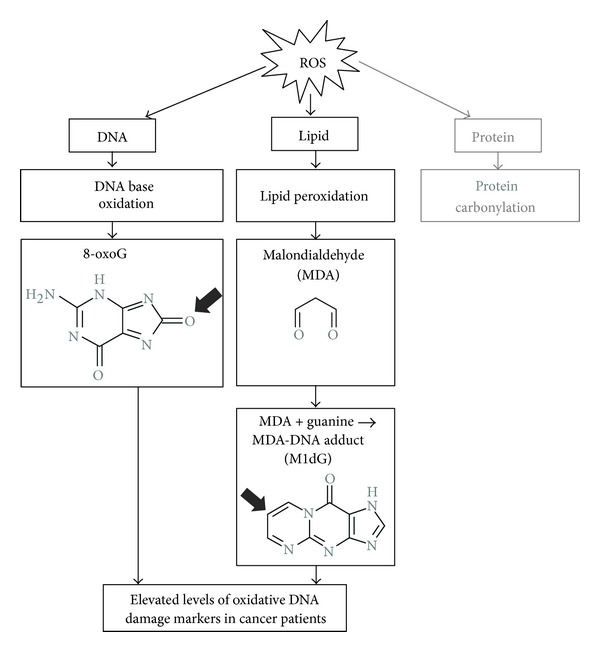
Markers of oxidative DNA damage are elevated in cancer patients. ROS causes oxidative damage to biomolecules such as DNA, lipids, and proteins, and the resulting end products are often detrimental to normal cell physiological functions. As the result of DNA base oxidation, 8-oxo-guanine (8-oxoG) can serve as a biomarker of primary oxidative DNA damage. When lipids are attacked by ROS, secondary DNA damage arises due to malondialdehyde (MDA), the end production of lipid peroxidation that can covalently bind to guanine and form MDA-DNA adduct (M1dG). In human cancer patients, both 8-oxoG and M1dG are found to be elevated, suggesting a correlation between higher oxidative stress and cancer.

Oxidative stress-induced lipid peroxidation is also associated with the early stages of carcinogenesis [10]. Malondialdehyde (MDA), the product of lipid peroxidation, can induce the formation of DNA adducts which leads to mutagenesis. In an epidemiological study of breast cancer, the level of the malondialdehyde-DNA adduct, 3-(2-deoxy-**β**-D-erythro-pentofuranosyl) pyrimido [1,2-*α*]purin-10(3*H*) one (M1dG), was significantly higher in breast tissue specimens from cancer patients than in those from healthy individuals [11]. Therefore, other than 8-oxoG, the level of M1dG has been employed as an indicator of cancer-associated oxidative DNA damage. These markers are used as measures of antioxidant activity in studies that assess the chemopreventive efficacy of anticancer agents including phytochemicals [9, 12, 13] (Figure 2).

## 2. Sources of ROS and Cellular Antioxidant Defense

ROS are genotoxic and ubiquitous. They include the superoxide anion radical (O_2_^∙−^), hydrogen peroxide (H_2_O_2_), the hydroxyl radical (OH^∙^), and the nitric oxide radical (NO^∙^) [14]. For maintenance of genome integrity and normal cell physiological function, cells have developed strategies to control ROS levels. Such control is known as antioxidant defense [14]. Cellular redox status, the level of ROS, is the net result of ROS arising from various origins and the capacity of the cell to remove it by antioxidant defense. Many preventive/therapeutic regimens, including those phytoagent-based, intervene in disease progression by fine-tuning the level of ROS and the corresponding antioxidant responses in the cell [15], and thus shifting the redox balance in favor of human health. Introductions of the various origins of ROS and cellular antioxidant defense mechanisms are outlined below.

### 2.1. Origins of ROS

**Figure 3:**
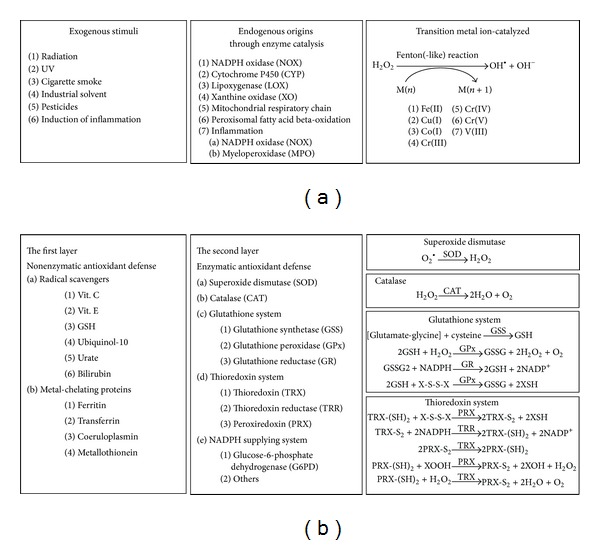
The source and clearance of ROS. (a) Three major origins of ROS. The sources of ROS can be roughly classified into three major categories: exogenous, endogenous, and transition metal ion-catalyzed. Exogenous sources of ROS can elicit radical chain reactions, contain/produce ROS, or stimulate enzymatic ROS production. Endogenous sources of ROS include the various enzymes that produce ROS as by-products or as signaling mediators or as antimicrobial agents during inflammation. Many of these enzymes can be activated by stimulation by cytokines and growth factors, such as NOX, LOX, XO, and MPO. Some CYPs are inducible and can be upregulated by environmental pollutants, dietary phytocompounds, or drugs. The transition metal ion-catalyzed Fenton-reaction produces highly reactive hydroxyl radical from hydrogen peroxide. (b) Layers of antioxidant defense. There are several layers of antioxidant defense. Basal level antioxidant defenses provide buffering capacity upon ROS challenge. Radical scavengers can directly quench ROS, and metal-chelating proteins can block ROS generation catalyzed by the Fenton or Fenton-like reactions. Further antioxidant capacity is provided by inducible antioxidant enzymes that are mostly under the regulation of Nrf2/ARE signaling (see Figure 4). ROS can oxidize the thiol group of amino acid residues leading to intermolecular or intramolecular disulfide bond formation. These disulfide bonds that are caused by oxidation can lead to structural/functional alteration of proteins. These disulfide bonds can be reduced by the glutathione system and the thioredoxin system allowing resumption of protein function. NADPH plays an indispensable role in the recycling of glutathione and thioredoxin, and therefore metabolic enzymes that are involved in NADPH generation also account for antioxidant defense.

Sources of ROS can be divided into three major categories: exogenous, endogenous, metal-catalyzed (Figure 3(a)). Exogenously, ROS levels are mainly increased by environmental and dietary factors. These factors may serve as prooxidants that elicit ROS directly through chemical reactions or through the inhibition of cellular anti-oxidant defense or as substrates or stimulators of ROS-producing enzymes. Environmental factors that increase ROS production include ultraviolet light, ionizing radiation, air pollutants, cigarette smoke, pesticides, and industrial solvents or chemicals. Dietary factors that induce ROS include food containing peroxidized lipids (from rotten oil), polycyclic aromatic hydrocarbons (PAH, from high-temperature processed hydrocarbon-based food), and food additives (preservatives).

Endogenously, ROS are generated during metabolic processes, such as mitochondrial oxidative phosphorylation, peroxisomal fatty acid beta-oxidation [16], catabolism of xenobiotics by cytochrome P450 monooxygenase (CYP) [17], purine by xanthine oxidase (XO) [18, 19], and lipid/fatty acid by cyclooxygenase (COX) [20, 21] or lipoxygenase (LOX) [22, 23]. Inflammation is another important endogenous source of ROS. During inflammation, ROS are generated via NADPH oxidase and myeloperoxidase which can protect against microbe or virus invasion; however, they might also be injurious to adjacent cells [24–27]. The positive feedback loop between oxidative insult, inflammation, and carcinogenesis is well recognized and appreciated as one of the hallmarks of cancer [28]. In metal-catalyzed generation of ROS, transition metal ions such as iron, copper, and chromium catalyze Fenton or Fenton-like reactions [29] that donate electrons and thus promote the production of hydroxyl radicals from hydrogen peroxide [30].

### 2.2. Cellular Antioxidant Defense Mechanisms: Control of ROS Levels and Repair of Oxidized DNA Bases

Proper control of ROS is critical for the maintenance of redox balance and genome integrity. Otherwise, excessive levels of ROS would overwrite the roles of ROS as signaling mediators and jeopardize the normal physiological processes inside the cell. Several layers of antioxidant defense have been proposed as preventive strategies against nucleic acid oxidation, including nonenzymatic removal of ROS by scavenger molecules, chelation of metals that catalyze ROS formation, inducible enzymatic removal of ROS, and the DNA repair system responsible for oxidative DNA lesion. Cellular molecules that can serve as radical scavengers form a first line of defense in the control of ROS levels (Figure 3(b)). These molecules include metabolites such as vitamin C, vitamin E, ubiquinol-10, and urate, as well as the tripeptide glutathione (GSH) and the thioredoxin (TRX) system [31]. Meanwhile, cellular metal-chelating proteins play key roles in controlling the level of free metal ions and thus enhance or prevent ROS generation by metal-catalyzed Fenton of Fenton-like reactions. These proteins include ferritin [32, 33], transferrin [34], coeruloplasmin [35], and metallothionein [36].

ROS scavengers and metal-binding proteins do not provide complete protection from ROS damage. Therefore, another layer of protection is provided in the form of enzymatic removal of ROS. Superoxide dismutase (SOD) is responsible for the transformation of superoxide anions into hydrogen peroxide, which is subsequently transformed into oxygen and water by catalase (CAT) or into water by glutathione peroxidase (GPx) [14]. The removal of hydrogen peroxide by GPx consumes the reduced form of glutathione (GSH) and generates the oxidized form (GSSG). GSSG can later be recycled by glutathione reductase (GR) and so replenish the GSH pool. Notably, metabolic enzymes responsible for NADPH production are critical factors in maintaining cellular redox balance, because NADPH is an indispensable factor responsible for the recycling of GSH and TRX by GR and thioredoxin reductase (TRR). Defects in NADPH supplying enzymes, such as glucose-6-phosphate dehydrogenase (G6PD) deficiency in humans, compromise recycling of glutathione and thioredoxin and so weaken antioxidant capacity and confer susceptibility toward oxidative insult [37]. SOD, CAT, GPx, GR, TRR, and NADPH producing enzymes together, therefore, increase the capacity of the cell to remove ROS through enzymatic means (Figure 3(b)).

**Figure 4:**
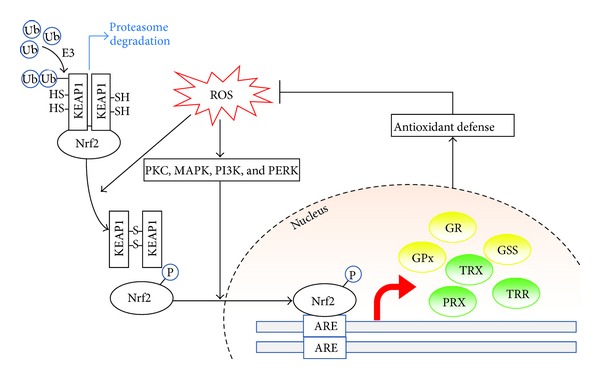
Inducible antioxidant defense regulated by Nrf2/Keap1 and the antioxidant response element. Under normal physiological conditions, the transcription factor Nrf2 is sequestered in the cytosol by Keap1. Keap1 recruits ubiquitin ligase E3 which then ubiquitinates Nrf2 and directs it to the proteasome degradation pathway. The increased level of ROS promotes the dissociation of Nrf2 and Keap1, either via activation of kinases that phosphorylate Nrf2 or by oxidization of key cysteine residues that govern Keap1 activity. The dissociated Nrf2 is then translocated into the nucleus and binds to the antioxidant response element (ARE). ARE-regulated genes are then transcriptionally activated, including a panel of antioxidant enzymes or proteins, such as glutathione synthetase (GSS), glutathione reductase (GR), glutathione peroxidase (GPx), thioredoxin (TRX), thioredoxin reductase (TRR), and peroxiredoxin (PRX). These inducible antioxidant enzymes then provide further ROS clearance capacity and antioxidant defense mechanism to exert a cytoprotective effect.

Cellular antioxidant defense is inducible and often up-regulated in response to oxidative stress or plant antioxidants. Cells sense and respond to changes in redox status by nuclear factor (erythroid-derived 2)-like 2 (Nrf2)/kelch-like ECH-associated protein 1 (Keap1) complex [38, 39], which when dissociated allows Nrf2 nuclear translation and binding to the antioxidant response element (ARE) to transactivate antioxidant enzymes and thus further elevate antioxidant capacity [40] (Figure 4). Under normal physiological conditions, transcription factor Nrf2 is sequestered in the cytosol by Keap1, which recruits ubiquitin ligase E3 that ubiquitinates Nrf2 and directs it to the proteasome degradation pathway. Increased levels of ROS promote the dissociation of Nrf2 and Keap1, either by the oxidization of key cysteine residues that govern Keap1 activity or via the activation of kinases (e.g., protein kinase C (PKC), mitogen activated protein-kinase (MAPK), phosphatidylinositide 3-kinases (PI3K) [41], and protein kinase (PKR-) like endoplasmic reticulum kinase (PERK) that phosphorylate Nrf2 [42]. The dissociated Nrf2 then translocates into the nucleus and binds to the ARE. ARE-regulated genes, such as glutathione synthetase (GSS), GR, GPx, TRX, TRR, and peroxiredoxin (PRX) are then transcriptionally activated [40]. These inducible antioxidant enzymes provide further ROS clearance capacity and thus confer cytoprotective effects ensuing Nrf2 activation in response to oxidative stress stimulation during inflammation [43] or in the presence of redox-modulating phytoagents [44, 45] (Figure 4).

**Figure 5:**
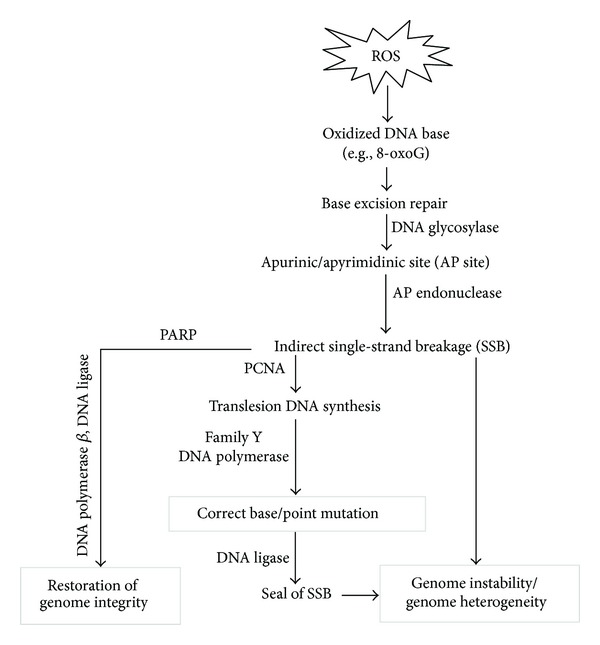
Repair of oxidative DNA damage introduces genome heterogeneity and instability. ROS causes oxidation of DNA bases which then elicit base excision repair machineries. First, the oxidized base is cleaved by glycosylase leaving an apurinic/apyrimidinic site (AP site). Second, the AP site is recognized by AP endonuclease that cleaves the phosphodiester bonds to remove the AP nucleotide and create the single-strand break (SSB) intermediate. DNA polymerase then resynthesizes the missing part of the DNA and later DNA ligase seals the nick. The low fidelity of the translesion DNA polymerase increases the chance of mismatched base-pairing and thus, leads to accumulation of point mutations which creates genome heterogeneity.

As nonenzymatic and enzymatic control of ROS levels cannot guarantee perfect/complete protection against ROS damage, oxidative damage continues to occur and accumulate in cells. To alleviate the negative effects elicited by oxidized biomolecules, especially DNA, cells have evolved sophisticated specific enzymatic repair systems. One such system, base excision repair (BER), repairs oxidized DNA bases (Figure 5) [5]. During BER, the oxidized base is first recognized and removed by DNA glycosylase leaving an apurinic/apyrimidinic (AP) site which is later recognized and cleaved by AP endonuclease on the phosphodiester backbone and leaves a DNA single-strand break (SSB) intermediate with a free 3′-OH end. Subsequently, PPAR binds to the SSB and recruits DNA polymerase *β* and DNA ligase which synthesizes the missing nucleic acid and seals the SSB to restore genome integrity. Nonetheless, PCNA, a DNA clamp protein that associates with and coordinates the DNA repair pathway, facilitates a DNA polymerase switch to the specialized Family Y DNA polymerase and increases the potential of generating point mutation. Family Y DNA polymerase carries out translesion DNA synthesis. The low fidelity of Family Y DNA polymerase introduces a higher frequency of mismatched base pairing than in regular DNA synthesis and therefore increases the incidence of point mutations [46, 47]. In the last step, DNA ligase seals the nick between the *de novo* synthesized nucleotide and adjacent nucleotides and completes the base excision repair process. The point mutations introduced during translesion DNA repair lead to genome heterogeneity between different cells and, chronologically, within the same cell (Figure 5).

## 3. “Double-Edged Sword” Role of Phytoagents as Redox Regulators in Cancer Management

### 3.1. Phytoagents in Cancer Management

Plants produce a remarkably diverse array of secondary metabolites (phytochemicals), many of which have evolved to combat microbial attack, resist environmental stress, or function as signaling molecules in interplant communication [48]. Human civilizations have used botanical preparations for treating and preventing various human diseases throughout history. Today, more than half of the anticancer drugs in clinical use are natural products or their derivatives and many are plant-derived phytochemicals [49, 50]. As cancer remains a major threat to health worldwide, there is global demand for more affordable and effective therapeutic alternatives. Moreover, concerns about drug resistance and the side effects of conventional therapeutic regimens currently used for cancer have renewed interest in phytochemicals derived from dietary foods and traditional medicines [51–55].

**Figure 6:**
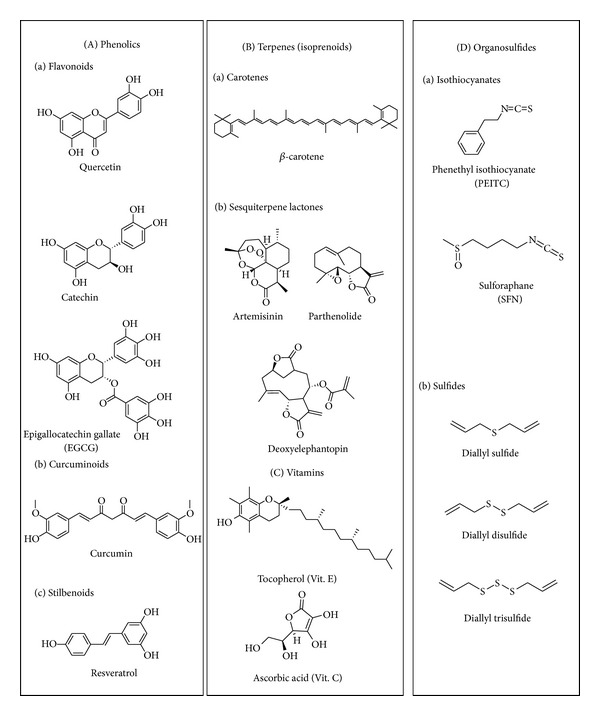
Representative phytocompounds with redox regulation capability. There are four major types of phytocompounds that can modulate intracellular redox status: (A) phenolics, (B) terpenes, (C) vitamins, and (D) organosulfides. They show free radical scavenging, Nrf2/ARE activation, and/or facilitation of ROS production in cancer cells.

The US National Cancer Institute (NCI) has identified more than 1,000 different phytoextracts or phytochemicals that possess cancer-preventive activity [15] and the components responsible for many of the cancer chemopreventive effects of various edible plants have been determined. For example, the cancer preventive effects of allium species (e.g., garlic) and cruciferous vegetables (e.g., broccoli and watercress) are attributed to organosulfur compounds (e.g., diallyl trisulfide) and isothiocyanates (e.g., sulforaphane (SFN) and phenethyl isothiocyanate (PEITC)), respectively [56]. Other naturally occurring phytochemicals found in fruits, vegetables, spices, herbs, beverages, and medicinal plants, such as resveratrol [57], genistein [58], curcumin [59], (–)-epigallocatechin gallate (EGCG) [60], and sesquiterpene lactones (e.g., deoxyelephantopin [61–63], artemisinin [64], and parthenolide) [65–67] have been reported to modulate multiple signaling cascades that are known to deregulate cancer cell activities [68]. Interestingly, these representative phytocompounds (Figure 6) exert their anticancer cell effects through modulating ROS activity and oxidative stress in cancer cells by antioxidant, pro-oxidant, or a dual as antioxidant and prooxidant under certain physiological or pathological conditions. The important dual, seemingly oppositional role of phytoagents as redox regulators involved in nucleic acid oxidation in cancer cells, is discussed below.

### 3.2. Phytoagents as Antioxidants for Cancer Prevention

In general, phytoagents with antioxidant properties are potentially useful in cancer prevention because they can protect healthy cells from oxidative DNA damage through direct radical scavenging, upregulation of antioxidant defense system, metal ion chelation, and/or additional anti-inflammatory activity. The latest developments in the evaluation of the antioxidant effects and related defense systems or molecular mechanisms of phytocompounds, with focus on oxidative DNA damage as a biomarker in cancer prevention, are discussed below.

#### 3.2.1. Major Antioxidant Mechanisms of Action of Phytoagents

*(a) Direct ROS Scavenging*. Phytoagents can attenuate ROS insults on biomolecules through direct scavenging of ROS. “Scavenging” refers to direct chemical modification of ROS and their stabilization by chemical reduction or electron-donation. In this way, the reduced form of a phytoagent molecule is consumed to buffer injurious ROS that might otherwise cause DNA damage. Phytoagents might have different scavenging capacity for different ROS and free radical species. For example, vitamin E and the carotenes have long polyunsaturated fatty acid chains, while vitamin C, flavonoids, and polyphenols have ring structures. They all share one structural commonality: conjugated systems, characterized by intermittent single bonds and double bonds which together form aligned p orbitals where pi electrons can move freely. The conjugated system can, therefore, donate electrons more easily and thus have high reducing capacity. This property gives these phytoagents ROS buffering capacity that protects important biomolecules from ROS attack.

*(b) Attenuation of the Fenton(-Like) Reaction by Direct Metal Ion Chelation.* Oxidative damage is one of the main forms of toxicity conferred by transition metal ions. In the Fenton(-like) reaction, the reduced form of a transition metal ion catalyzes the generation of the highly reactive hydroxyl free radical from hydrogen peroxide. Therefore, the more free form transition metal ions there are, the more hydroxyl free radical formation occurs by the Fenton(-like) reaction, and the more serious the oxidative damage to biomolecules including DNA. Will be Phytoagents can attenuate Fenton(-like) reaction by reducing the level of transition metal ion. Through direct chelation by phytocompounds containing a catechol or galloyl structure, transition metal ions are sequestered from solution and therefore prevented from participating the Fenton(-like) reaction [69, 70]. This is another indirect way by which phytoagents exert antioxidant effects.

*(c) Induction of Antioxidant Response Element-Controlled Genes through Nrf2 Activation.* Dietary levels of phytochemicals have been suggested to trigger induction of low levels of oxidative stress that may “prime” cellular antioxidant defense systems to resist higher levels of oxidative insults thus offering protection against carcinogenic insult [60]. These types of phytochemicals might have little antioxidant effect *in vitro* in terms of ROS scavenging capacity; nonetheless, in some cases, they activate the master transcription factor Nrf2 which governs the expression of a set of antioxidant-related genes. Therefore, through activation of Nrf2 and the subsequent up-regulation of endogenous antioxidant defense, these phytochemicals confer antioxidant effects in an indirect way.

Phytoagents from various structural categories have been shown to activate Nrf2 with varied potency [71]. In general, phytoagents with electrophilic groups that are thiol-reactive induce the most potent Nrf2 activation when compared based on fold of induction of Nrf2-regulated NADPH: quinone reductase [72]. Some phytoagents without electrophilic groups could also induce Nrf2, though to a lesser extent. These types of phytoagents might activate Nrf2 indirectly through modulating signaling pathways whereas thiol-reactive electrophiles can directly modify the redox-sensitive cysteine residues in the Nrf2/Keap1 complex thereby promoting the dissociation of the complex and the nuclear translocation of Nrf2.

*(d) Attenuation of Inflammation through Inactivation.* NF-*κ*B is the master transcription factor that governs the expression of many inflammation-related genes. Notably, the activation of NF-*κ*B is redox-sensitive. High endogenous ROS level stimulates NF-*κ*B activation, which then leads to a pro-inflammatory response and further exacerbates the intracellular redox status [73–77]. Such a feedback loop mediated by redox-sensitive NF-*κ*B activation often leads to chronic inflammation, one of the hallmarks of cancer. Many phytoagents exhibiting an anti-inflammatory effect have been shown to efficiently suppress NF-*κ*B activation. Suppression of NF-*κ*B can be achieved by either the aforementioned antioxidant actions or through direct chemical modification of NF-*κ*B redox-sensitive cysteine residues by phytoagents with electrophilic groups, such as C=O, N=C=S or organosulfide groups to compromise its ability to translocate to the nucleus and bind DNA.

#### 3.2.2. Antioxidant Effects and Defense Systems of Selected Phytoagents

Vitamins and phenolics (two well-known groups of antioxidants), as well as electrophilic phytocompounds, are used below to exemplify the latest developments in the evaluation of the antioxidant effects and related defense systems of phytocompounds with a focus on oxidative DNA damage as a biomarker in cancer prevention.

*(a) Vitamins.* The ability of macronutrients and micronutrients present in fruits and vegetables to reduce the risk of cancer is well known. Among these compounds, the antioxidant vitamins and their precursors have been extensively studied [15]. Vitamin C (ascorbic acid), vitamin E, and *β*-carotene are often referred to as “antioxidant vitamins.” Vitamin C cooperates with vitamin E to generate *α*-tocopherol from *α*-tocopherol radicals in membranes and lipoproteins. Through working along with other antioxidant enzymes, these antioxidants have been suggested to reduce oxidative damage in humans [78], and thereby minimizing the risk of certain chronic diseases [79–81]. However, early epidemiological studies and clinical trials investigating the efficacy of these vitamins in affecting disease outcome concluded that there was insufficient evidence to link supplementation of humans with vitamin C, vitamin E, or *β*-carotene with a reduction in *in vivo* oxidative damage to lipids, proteins, or DNA based on the measurement of oxidative biomarkers [82]. More recent clinical trials also suggest no correlatable effect between individual vitamins and chemoprevention [83, 84]. Further, anticancer properties reported for different vitamins have been discrepant. The history of the most well-known antioxidant, vitamin C, in cancer treatment is controversial while vitamins A and E only showed dispensable effects in tumor elimination [85]. However, the role of vitamin D in cancer treatment and prevention is promising [86, 87]. Interestingly, a large-scale, randomized, double-blind, placebo-controlled trial in male physicians showed that, compared with placebo, men taking a daily multivitamin had a statistically significant reduction in the incidence of total cancer; however, there was no significant effect on some specific cancer types, such as prostate cancer and colorectal cancer. It was therefore concluded that “daily multivitamin supplementation modestly but significantly reduced the risk of total cancer [88].”

Recently, in a large cohort study with 356 healthy subjects, dietary intake of vitamins was demonstrated to be associated with reduced levels of markers of DNA damage and oxidation (M1dG and 8-oxoG) measured in peripheral white blood cells. Notably, the associations were stronger in nonsmokers than in smokers [89]. It is important to keep in mind that several environmental factors can affect the antioxidant capacity of these vitamins. Environmental factors such as smoking and metal intoxication that causes excessive ROS burden to the body should be avoided, because antioxidant phytoagents can prevent *de novo* oxidation to nucleic acid but are not able to rescue or reverse oxidized nucleic acid caused by persistent oxidative insults from environmental stimulation. In another study, the protective effects of vitamin C and a natural phenol resveratrol on ethanol-induced oxidative DNA damage in human peripheral lymphocytes were investigated. Resveratrol showed significant DNA protection in a 24 h experiment, while the protective effect of vitamin C was seen in only 1 h. Both compounds were shown to directly scavenge hydroxyl radicals produced during ethanol metabolism. In addition, resveratrol inhibited dehydrogenase gene expression and activated the base excision repair (BER) system, mechanisms which may underlie its substantial effect on DNA protection. Vitamin C, however, showed no effect on the ethanol metabolic pathway or the BER system [90]. The antioxidant properties of vitamins in comparison to whole fruits and vegetables as anticancer agents are also of interest. The effectiveness of kiwifruit in decreasing oxidative DNA damage was assessed using comet assay (single-cell gel electrophoresis) to measure damage to lymphocytes collected from a human trial in which subjects drank kiwifruit juice. It was observed that a simple extract of kiwifruit was more effective than a solution of vitamin C in protecting DNA from damage *in vitro* [91]. This study demonstrated that the significant antioxidant activity of kiwifruit *ex vivo* and *in vitro* is not attributable entirely to vitamin C contained in the fruit. Instead, other components like phenolics and vitamin E may also contribute to the antioxidant effect of kiwi fruit extract [92]. These studies suggest an undetermined role of vitamin C present in fresh fruits, although different vitamin C content present in kiwifruit extract might result in different protective effects.

*(b) Phenolics*. Phenolic compounds are present in high concentrations in many components of the so-called “Mediterranean diet,” including fruit and vegetables. These compounds seem to scavenge ROS, resulting in protection against oxidative DNA. This assumption was verified by testing the effect of Mediterranean plant extracts (*Crepis vesicaria* L, *Origanum heracleoticum*, *Scandix australis* L, *Amaranthus *sp., *Scolymus hispanicus* L, and *Thymus piperella* L) on oxidative DNA damage induced in lymphocytes by H_2_O_2_ in relation to their polyphenolic content using comet assay [93]. This study revealed that the protection of DNA by phenols present in Mediterranean plants is only partly due to ROS scavenging properties. Phenols can also attenuate Fenton(-like) reactions through metal ion chelation and induce endogenous antioxidant defense through Nrf2 activation. Apparently, ROS scavenging only partially contributes to antioxidant activity of Mediterranean diet-derived phenolics or other phytochemicals. Their protection against oxidative DNA may involve other redox regulation such as upregulation of antioxidant enzymes in cells and attenuation of Fenton(-like) reaction by metal ion chelation.

In the carcinogenesis of hepatocellular carcinoma (HCC), oxidative stress is a major predisposing condition which is relevant to the development and progression of the cancer. In search for a dietary chemopreventive approach for the lethal HCC, pomegranate, an ancient fruit has gained attention owing to its significant antioxidant properties mainly contributed by the anthocyanins and ellagic acid derivatives [94, 95]. Pomegranate emulsion, a proprietary combination of aqueous phase extract and pomegranate seed oil containing several polyphenolic compounds, mixed with octadecatrienoic acids, sterols, steroids, and *γ*-tocopherol, was found to prevent hepatocarcinogenesis through induction of Nrf2-regulated phase II xenobiotic-metabolizing genes such as several GST isozymes that are involved in antagonizing oxidative stress [96]. A similar Nrf2-mediated antioxidant effect was also observed in HCC rats treated with blackcurrant anthocyanins [97].

Flavonoids are naturally occurring diphenylpropanoids that appear in animal and human cells following consumption of vegetables, fruits, and beverages such as tea and wine. Flavonoids can be classified into six major subgroups: flavonols (e.g., quercetin, kaempferol), flavones (e.g., apigenin, luteolin), flavanones (e.g., hesperidin, naringenin), flavan-3-ols (e.g., catechin, theaflavin, and gallic esters of catechin and theaflavins), anthocyanidins (e.g., pelargonidin, cyanidin), and isoflavones (e.g., genistein, daidzein). Epidemiological studies suggest that dietary intake of flavonoids may reduce the risk of tumors of the breast, colon, lung, prostate, and pancreas. However, the generalizability of these anticancer effects remains a subject of study [98]. 

*(c) Electrophilic Phytochemicals.* Electrophilic phytochemicals, such as phenethylisothiocyanate (PEITC), sulforaphane (SFN), turmeric, curcumin, and EGCG, prevent oxidative modification and mutation of genes through activation of the Nrf2/Keap1 complex [45, 99–101]. These phytochemicals modulate Keap1-associated transcriptional regulation which results in up-regulation of ARE-bearing genes encoding phase II detoxifying enzymes and transporters that protect normal cells from ROS, reactive nitrogen species (RNS) or reactive metabolites of carcinogenic species [71]. Such responses are thought to represent a form of cellular adaptation to chemicals and oxidative stress that maintains cellular redox homeostasis [15, 99]. Therefore, the use of dietary phytochemicals to regulate Nrf2-dependent antioxidant response to counter oxidative DNA damage has emerged as a promising strategy for cancer prevention.

Hormonal factors, especially 17ß-estradiol (E2), play a major role in the etiology of breast cancer where the circulating levels of E2 itself are an independent risk factor. E2 can cause both oxidative DNA damage and attenuate DNA repair leading to oncogenic mutagenesis [102]. In the liver, the metabolism of E2 to its various phase I metabolites, such as the carcinogenic 4-hydroxy estradiol (4E2), primarily involves the cytochrome P450 enzymes CYP1A2 and CYP3A4 [103]. Dietary berries and their chemical constituents are known for their cancer preventive potential, which were recently shown to affect the enzymes involved in carcinogen metabolism in mouse liver [104] and significantly reduced hepatic oxidative DNA damage, indicated by the level of 8-oxoG and other polar adducts validated by P^32^-postlabeling experiments. Compared to crude berry juices, ellagic acid, one of the bioactive components found in berries, showed more elimination of oxidative DNA adducts induced by redox cycling of 4E2 catalyzed by copper chloride *in vitro* [105].

### 3.3. Phytocompounds as Prooxidative Agents for Cancer Therapy

Prooxidant phytoagents, on the other hand, are particularly effective in treating aggressive tumors with abnormally radical-reactive cellular environments. They act by tipping the limit of oxidative stress that can be tolerated by tumor cells over a limit, thus triggering apoptosis and cell death [106]. Although pro-oxidant effects are observed after treatment with certain phytoagents, generally, phytoagents do not produce ROS directly. Instead, their prooxidant effect is highly dependent on the original redox status of the cell which determines sensitivity to cytotoxicity mediated by phytoagents. The basal redox levels of cancer cells are different from those of normal cells. Higher levels of free form metal ions and higher levels of endogenous ROS production in cancer cells sensitizes them to phytoagent-mediated prooxidant cytotoxicity [30, 107, 108]. In this section, we elaborate on how phytoagents act as prooxidants to selectively kill cancer cells and their effects in cancer chemotherapy.

#### 3.3.1. Major Prooxidant Mechanisms of Action of Phytoagents

*(a) Promotion of Fenton(-Like) Reactions by Catalyzing Redox-Cycling of Metal Ions*. Phytoagents with strong reducing capacity can reduce not only ROS but also metal ions. Under normal physiological conditions, most metal ions are complexed with proteins and few exist in free form. However, in the presence of abundant free form metal ions, phytoagents catalyze Fenton(-like) reactions that produce injurious hydroxyl radicals [29, 109]. Notably, cancer cells develop abnormally high concentrations of metal ions due to overexpression of the transferrin receptor [110, 111]. When excessive concentrations of free form metal ions exist, classical antioxidant phytoagents catalyze the redox cycling of metal ions by reducing their oxidized form. As a result, a burst of hydroxyl free radical production ensues and the phytoagents become pro-oxidants.

*(b) Basal ROS Generation through Glutathione Depletion by Electrophiles*. Phytoagents with electrophilic groups can form covalent bonds with cysteine resides of proteins. Glutathione, the most abundant cysteine-containing peptide, thus can be rapidly depleted due to adduct formation with electrophilic phytoagents [112–115]. Upon glutathione depletion, the buffering capacity of ROS is attenuated so that the basal ROS production is revealed. Therefore, electrophilic phytoagents exhibit pronounced pro-oxidant effect in cancer cells with high ROS production and push cancer cells over the tolerable limit of ROS. In contrast, the same dosage of phytoagents produces a negligible pro-oxidant effect in normal cells with low basal ROS production and boosts antioxidant response by Nrf2 activation [71, 100, 116–121].

#### 3.3.2. Prooxidant Effects and Defense Systems of Selected Phytoagents

ROS and cellular oxidative stress have long been associated with cancer [122]. Hypoxic condition, that is, low ambient oxygen pressure, is well described in cancer cells, particularly in the central area of the tumor nodule or mass [123]. These cancer cells act more like anaerobic bacteria, showing low levels of mitochondrial oxidative phosphorylation, and generally survive through the generation of ATP in an oxygen-independent manner [124]. Many conventional anticancer drugs including vinblastine, doxorubicin, campthotecin, cisplatin, and inostamycin have been reported to activate a caspase-3(-like) protease causing generation of H_2_O_2_ presumably through the activation of NADPH oxidase that subsequently induces apoptosis in cancer cells [125]. Intriguingly, cancer cells are frequently deficient in crucial antioxidative enzymes, such as catalase, GPx, and SOD, and therefore demonstrate a high vulnerability to ROS. One antitumor strategy is to deliver excess oxidative stress into tumor cells or to target the disruption of the antioxidative defense systems of tumor cells. This strategy has been termed “oxidation therapy” in cancer treatment [126]. Several studies have reported that certain dietary anticancer/cancer preventive agents cause generation of ROS specifically in tumor cells, not in normal cells [56, 127, 128]. Through adaptation, normal cells that are exposed to pro-oxidant chemopreventive agents which generate oxidative stress can acquire resistance to transformation via adjusting the normal redox tone of these cells. In contrast, transformed cells, which typically endure an oxidizing intracellular environment, would ultimately succumb due to an excess of ROS caused by the same agent. ROS and cellular redox tone are exploitable targets in cancer chemoprevention via the stimulation of cytoprotection in normal cells and/or the induction of apoptosis in malignant cells [129]. Dietary intake of such chemopreventive agents could be a prefect strategy to achieve this purpose.

*(a) Sulfur-Containing Compounds*. Diallyl disulfide (DADS) and diallyltrisulfide (DATS) which are found in abundance in garlic are among the dietary factors studied extensively for their anticancer action involving induction of oxidative stress in the human body, as reviewed elsewhere [130]. The pro-oxidant and thiol-adducting activities of these electrophilic organosulfur compounds are attributed to their reactive isothiocyanate (R–N=C=S) pharmacophore. Dietary isothiocyanates include sulforaphane, phenethyl isothiocyanate (PEITC), benzyl-isothiocyanate, and 6-methylsulfinylhexyl-isothiocyanate (Figure 6). Originally, copper-mediated oxidative DNA damage induced by these isothiocyanates was considered to be carcinogenic [131]; however, later studies demonstrated that these phytochemicals exhibit preferential cytostaticity in premalignant and tumor cells via their pleiotropic pro-oxidant activities as reviewed elsewhere [106].

*(b) Curcumin*. Curcumin (diferuloylmethane) from turmeric, like isothiocyanates, is a pleiotropic redox modulator that is involved in multiple cellular activities as a pro/antioxidant and metal chelator, as recently reviewed [59]. Curcumin, which contains an electrophilic Michael acceptor as an active moiety, can also mediate strand scission of DNA in the presence of Cu (II) [132]. The compelling anticancer activities of curcumin have been widely demonstrated across different cancer cell lines and animal systems, as a function of abovementioned reactive pharmacophores targeting various cellular molecules. Currently, the cancer preventive/therapeutic potential of curcumin, as single or combinatorial agent, is under evaluation in various clinical trials, including multiple myeloma, rectal cancer, metastatic colon cancer, advanced osteosarcoma, and pancreatic cancer [59].

*(c) Sesquiterpene Lactones*. The sesquiterpene lactones (SLs) have also gained considerable attention for their effectiveness in treating inflammation, headaches, infections, and other human diseases. SLs contain Michael acceptors that act as electrophiles that can increase cellular ROS and modulate specific redox sensitive targets in cancer cells. Artemisinin and parthenolide (Figure 6) are SL-derived drugs now being evaluated in cancer clinical trials [133–138]. Artemisinin, isolated from *Artemisia annua* (qinhao, sweet wormwood), possesses an endoperoxide bridge in the reactive pharmacophore that can be activated and cleaved by endogenous ions, leading to the generation of radical species and ROS through the Fenton reaction, which was observed to be a common mechanism underlying both the antimalarial and anticancer activities of the compound [139]. Parthenolide, identified from feverfew (*Tanacetum parthenium*), contains an electrophilic *α*-methylene-*γ*-lactone as the active moiety underlying its anticancer activity related to the Michael acceptor electrophile [66, 67]. Phytochemicals with prooxidant properties such as the SLs with Michael acceptor electrophiles have the potential to sensitize tumors in cancer treatment. For example, concurrent delivery of the SL parthenolide and the clinical drug paclitaxel in mixed micelles greatly improved the therapeutic response of resistant lung cancer cell lines to paclitaxel treatment [140]. In a mouse peritoneal dissemination model, parthenolide also improved the chemosensitivity of paclitaxel against gastric cancer through deregulation of the NF-*κ*B signalling pathway [141]. Nevertheless, parthenolide and dehydrocostus lactone can also suppress cancer cell activity through downregulating other molecular targets, such as mitogen-activated protein kinase (MAPK) and protein kinase C, and induction of c-Jun-N-termial kinase (JNK) [142].

In our laboratory, we identified a germacranolide SL deoxyelephantopin (DET) from a medicinal plant *Elephantopus scaber *(Asteraceas) which contains an *α*-methylene-*γ*-lactone, an *α*,*β*-unsaturated lactone and a methacrylate ester side chain [62]. DET could induce ROS in breast cancer cells which became the upstream stimulus for the formation of centrosomal ubiquitinated protein aggregates and the induction of protein carbonylation that might subsequently restrict cancer cell motility [63]. DET was also observed to activate ER stress- and JNK pathway-mediated apoptosis in mammary carcinoma cells triggered by ROS [62]. However, it is not yet clear whether DET caused oxidative DNA damage through the involvement of transition metals. Illustration that the anticancer activity of DET, the same as artemisinin, is through its role as a pro-oxidant suggests that pro-oxidant intervention using SLs may constitute a promising anticancer strategy.

### 3.4. Cancer-Associated Transition Metals in Phytochemical-Mediated Redox Regulation

**Figure 7:**
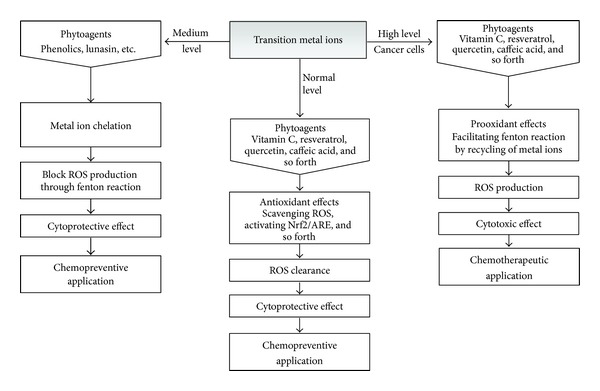
Role switches under different conditions—phytoagents function as both antioxidants and prooxidants in concert with transition metal ions. The level of transition metal ions determines whether a phyto-antioxidant ultimately functions as cytoprotective antioxidant or cytotoxic prooxidant. Under normal levels of transition metal ions, phytoantioxidants serve as radical scavengers and Nrf2/ARE activators that confer a cytoprotective effect that can be applied in chemoprevention. When the level of intracellular transition metal ion is high, such as in cancer cells, phytoantioxidants recycle the metal ions and thus facilitate ROS production through the Fenton or Fenton-like reactions. Otherwise, metal ions catalyze the cleavage of phytoagents and generate radical cleavage products that can cause ROS. Such a prooxidant effect further drives the redox-sensitive cancer cells to their antioxidant limit and leads to cytotoxicity that can be applied as a chemotherapeutic strategy. On the other hand, metal-chelating phytoagents reduce metal ion levels and thus block the ROS producing Fenton(-like) reaction and provide a cytoprotective effect.

Several essential transition metals, such as zinc, iron, copper, cobalt, and manganese, are known to regulate various metabolic and signaling pathways. For example, iron is an essential element in oxygen transportation [143] while copper is an essential component of several antioxidant enzymes [144]. In cancer cells, high metal ion concentration is one factor that contributes to the observed high base level of oxidative stress, which raises the possibility of killing cancer cells by dosing with metal supplements [145]. However, the prooxidant effect of metal ions is also known to initiate carcinogenesis [30], which raises concerns about applying metal supplementation as a therapeutic strategy against cancer. However, some studies indicated that cancer cells are prone to proliferate in environments with high levels of copper and iron and therefore suggested that these ions maybe be functionally involved in carcinogenesis [146, 147]. In a national cohort of the United States adults, serum concentrations of iron and copper were shown to correlate with mortality rate in cancer patients [148]. Due to the significant role of these metal ions in cancer epidemiology, their levels in different cancers were reviewed by Gupte and Mumper [145]. In comparison to normal individuals, the Cu (Zn, Se, Fe) ratios are usually higher in patients suffering from breast [149], cervical [150], ovarian [150], lung [151], prostate [152], bladder [153], and stomach cancer [154], and leukemia [155]. Increased levels of copper have also recently been correlated with poor survival in breast cancer patients [156]. The major metal ion contained in chromatin, copper is closely associated with the DNA bases, especially guanine [157]. As one of the redox active metals, copper can directly catalyze the formation of ROS via the Fenton reaction and cause oxidative stress in the cells [158]. The intracellular level of transition metal ions can determine whether phytoantioxidants act as cytoprotective antioxidants or cytotoxic prooxidants.  Figure 7 summarizes the current understanding of the interplay between phytoagents and transition metal ions and the antioxidant/pro-oxidant role switch of phytoagents in response to the level of metal ions. The level of transition metal ions determines whether a phytoagent ultimately functions as cytoprotective antioxidant or cytotoxic pro-oxidant. Under normal level of transition metal ions, phytoantioxidants serve as radical scavengers and Nrf2/ARE activators that confer a cytoprotective effect that can be applied in chemoprevention. When the level of intracellular transition metal ion is high, such as in cancer cells, phytoagents recycle the metal ions and thus facilitate ROS production through the Fenton or Fenton-like reactions. Otherwise, metal ions catalyze the cleavage of phytoagents and generate radical cleavage products that can cause ROS. Such a prooxidant effect further drives the redox-sensitive cancer cells to their antioxidant limit and leads to cytotoxicity that can be applied as a chemotherapeutic strategy. On the other hand, metal-chelating phytoagents reduce metal ion levels and thus block the ROS producing Fenton(-like) reaction and provide a cytoprotective effect.

#### 3.4.1. Ion Chelation by Phytoagents

Increasing numbers of studies are evaluating the antioxidant properties of phytochemicals through assessment of their ability to chelate metal ions that lead to attenuated reactivity of free radicals. Water extracts of pine needles inhibited oxidative DNA damage probably due to their strong hydroxyl radical and intracellular ROS scavenging activity and the chelating action of the iron (Fe2+) ion [159]. Antioxidant activity was reported for lunasin, a novel preventive peptide purified from *Solanum nigrum* L, which is also found in soy, barley, and wheat. The peptide did not scavenge endogenous hydroxyl radicals but inhibited the Fenton reaction by chelating iron ions, thus protecting DNA from oxidative damage [160]. The antioxidant properties of phenolic compounds are clear; however, the contribution of metal ion chelation to the antioxidative effect of these compounds is not yet conclusive. One study showed that the orthodihydroxy polyphenols bearing catechol or galloyl groups exhibit strong metal chelating activity [161]. In the study by Andjelkovic and colleagues, the ability of the phenolic compounds which chelate iron was ranked based on iron binding constants in ascending order. Protocatechuic acid was the weakest chelator, followed by hydroxytyrosol, gallic acid, and caffeic acid, with chlorogenic acid as the strongest chelator [162]. Iron chelation by phenolic compounds, phytochemicals in pine needle extracts, or by the peptide lunasin, which subsequently inhibited DNA oxidation, may deserve further exploration for their potential in cancer prevention.

The reactivity of metal ions can be attenuated indirectly through inhibition of their transportation. Dihydroartemisinin was reported to decrease iron uptake and disturb iron homeostasis in cancer cells through down regulating cell-surface transferrin receptor-1, which may be a novel mechanism of dihydroartemisinin independent of oxidative damage that has been previously mentioned as anticancer property of artemisinin [163]. The disturbance of iron homeostasis in cancer cells via irondepletion by natural or synthetic iron chelators has recently been shown to inhibit tumor growth by therapeutically manipulating iron level [164]. The effect of phytocompounds on deregulation of reactive ion metabolism in tumor cells is worth further exploration.

It is interesting to note that a prokaryotic glutathione analog, namely, ergothioneine, can protect cells from oxidative damage as measured by 4-HNE and partially rescue cell death caused by irradiation [165]. Another report showed that ergothioneine forms a chelation complex with copper and therefore protects cells from copper-induced DNA damage [166].

#### 3.4.2. Transition Metal-Mediated Prooxidant Properties of Phytochemicals in Anticancer Activity

Under certainconditions, antioxidants can act as prooxidants [167]. Caffeic acid produces hydrogen peroxide which is activated by transition metals to cause oxidative DNA damage *in vitro *and in cultured human cells in the presence of Mn(II) or Cu(II) [168]. In another study using DNA fragments isolated from the human *p53 *gene, quercetin increased 8-oxoG levelsignificantly in the presence of copper ions (Cu^2+^), whereas 8-oxoG formation by kaempferol or luteolin was insignificant [169]. These early studies raised concern about whether ingestion of these phytochemicals may lead to increased risk of cancer. Lately, rats treated with 7,12-dimethylbenz[a]anthracene (DMBA) have become a widely used model for mammary carcinogenesis and in recent study, dietary supplementation with copper alone or together with the grape polyphenol resveratrol was found to promote carcinogenesis through increased frequency of microsatellite instability [170]. Later, a similar result was observed in the DMBA-model treated with combined supplementation with zinc ions and resveratrol [171]. However, a different mechanism was reported for resveratrol action in another cancer model with different stage of carcinogenesis. Resveratrol and its derivatives increase copper-mediated oxidative DNA damage by their pro-oxidant properties coupled with higher apoptosis induction in human leukemia cell lines [172].

The well-known antioxidant vitamin C, for example, was also found to act as a pro-oxidant *in vitro *when mixed with transition metal ions [173]. In healthy humans, Rehman and colleagues observed an increased level of oxidative DNA damage after 6-week supplementation of a mixture of ferrous sulphate andvitamin C, suggesting that this combination acts as a pro-oxidant; however, a longer period of supplementation by 12 weeks did not show significant effect [174]. Intriguingly, catalytic therapy that involves hydroxyl radical induction through a redox active mixture of vitamin C/medicinal herbal extracts and copper has been employed to improve the treatment of cancer patients [175, 176]. The Bhat group that established a model that involves human peripheral lymphocytes and comet assay carried out a series of studies on plant-derived polyphenolic antioxidants and proved that the mechanism is not restricted to vitamin C [177–179]. The most recent finding from the group is that the polyphenolic compound gossypol from the cotton plant and its derivative apogossypolone also cause oxidative damage to DNA by mobilizing endogenous copper in lymphocytes [180]. Although the reported mechanism was mainly the result obtained from lymphocytes; nevertheless, it could imply the anticancer property of polyphenols based on the abundant copper detected in different types of tumors [145, 153]. The enhanced electron transfer between transition metals and phytochemicals probably occurs in cancer cells with higher levels of copper ions, which may induce ROS generation subsequently leading to DNA damage [178, 180].

However, the mixture of a polyphenol and a transition metal was shown to promote tumor growth in mice with carcinogen induction that mimics the process of cancer initiation [170, 171]. These studies raise concerns about the potential carcinogenic activities of phytoagents. It is not clear whether the mixture of antioxidant phytochemical and transition metal resembles the oxidative stress that could possibly initiate tumorigenesis in normal cells, but that such a prooxidant effect drives the redox-sensitive cancer cells to their antioxidant limit and leads to cytotoxicity that has been applied in catalytic therapy. More studies are required to clarify the interaction of phytoagents and redox active metals as their oxidative potential may initiate tumors in a healthy individual.

## 4. Future Prospects

**Figure 8:**
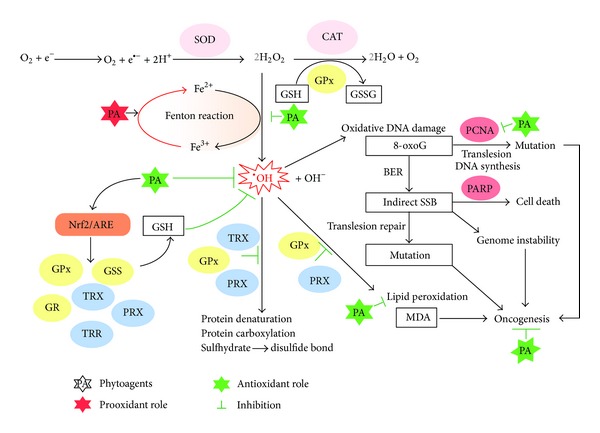
Summary of mechanisms of action of phytoagents in chemoprevention and chemotherapeutics through modulating oxidative stress. In the presence of ferrous ions (or other transition metal ions), phytoagents recycle the metal ion and thus promote the Fenton reaction that generates the highly reactive hydroxyl radical from hydrogen peroxide. Such prooxidant effects of phytoagents in the presence of metal ion can overwrite their cytoprotective roles because the production of ROS may be faster than the induction of antioxidant defense. Hydrogen peroxide imposes oxidative damage on biomolecules, such as proteins, lipids, and DNA, and leads to protein carbonylation, lipid peroxidation, and DNA base oxidation, which can be prevented by phytoantioxidants. Phytoantioxidants can activate Nrf2/ARE signaling and thus transcriptionally upregulate a panel of antioxidant genes that can provide further antioxidant capacity. Glutathione synthetase (GSS) can raise the level of glutathione (GSH) which can reduce oxidative damage by scavenging hydroxyl radicals, which otherwise cause oxidative DNA damage and increase the chance of point mutation and genome instability during the DNA repair process while glutathione reductase (GR) recycles the oxidized form of GSH and maintains the level of the reduced form of GSH. Glutathione peroxidase (GPx), thioredoxin (TRX), and peroxiredoxin (PRX) can prevent oxidative insults on proteins and lipids.

This review provides updated and integrative information about the regulation of nucleic acid oxidation by phytoagents in cancer. Animal models and human epidemiological studies have revealed that phytochemicals prevent carcinogenesis through direct ROS scavenging or induction of cellular antioxidant defense systems that consist of detoxifying enzymes, defense machinery mediated by Nrf2-antioxidative stress, and inhibiting inflammatory signaling pathways that together protect cells from DNA damage by ROS and reactive metabolites of carcinogens [42, 57, 58] (Figure 8). Investigation of oxidative modulation of proteins and lipids as well as DNA by phytochemicals provides a comprehensive picture of their functions as redox regulators in cancer. In general, antioxidant phytoagents are potentially useful in cancer prevention because they can protect healthy cells from oxidative DNA damage through radical scavenging, antioxidant defense system stimulation, and metal ion chelation; prooxidant phytoagents, on the other hand, are particularly effective in treating aggressive tumors with abnormally radical-reactive cellular environments by exceeding the limit of oxidative stress that can be tolerated by tumor cells. Cancer cells, in general, have a higher basal redox level due to either defects in antioxidant defense or increased production of ROS during oncogenic transformation [122, 126]. Therefore, when challenged with similar quantities of ROS, cancer cells fail to buffer/clear excessive ROS and cell death ensues. In contrast, normal cells with lower redox levels are capable of buffering/clearing ROS by inducible antioxidant defense regulated by Nrf2/ARE signaling and are thus protected from cell death. Recently, dietary levels of phytochemicals have been suggested to trigger induction of low levels of oxidative stress that may ‘‘prime" cellular antioxidant defense systems to resist higher level of oxidative insults, thus offering greater protection against carcinogenic insult [60].

However, several studies have also hinted at a “dark” side of these cell-protective mechanisms. For example, the cytotoxicity of the anticancer drug platinum was attenuated by base excision repair of ROS-induced formation of 8-oxoG, indicating that repairing genotoxic damage could contribute to multidrug resistance of cancer cells [181]. Restoration of glutathione level by overexpression of *γ*-glutamylcysteine synthetase was found to prevent DNA damage and subsequent apoptosis caused by genotoxic agents in a resistant human ovarian carcinoma cell line [182]. Overexpression of catalase was found to cause drug resistance in breast cancer cell lines in chemotherapy [183]. These findings imply that alteration of the expression of antioxidant enzymes could be a mechanism through which cancer-cell resistance to redox-based chemotherapeutic agents is promoted. On the other hand, several phytochemicals have been indicated to upregulate the Nrf2 pathway which stimulates the defense system and leads to cancer prevention. However, overexpression of Nrf2 and its downstream genes was also observed in several cancer cell lines and human tumors, rendering cancer cells at an advantage for survival and unlimited proliferation. In addition, increased Nrf2 activity was found in some resistant cancer cells; in other words, to overcome chemoresistance in tumors, the Nrf2 pathway has to be deregulated [184]. Therefore, sophisticated design is necessary and caution has to be taken when administrating and handling Nrf2-dependent (as discussed above) phytochemicals in cancer patients, given that transformed cancer cells that are “overprotected” by antioxidants could possibly develop drug resistance.

Nrf2 is one of the key players in phytoagent-mediated antioxidant defense whose activation confers a chemopreventive effect. However, recent studies indicate that Nrf2 itself also plays a double-bladed-sword role in cancer management [185]. On one hand, Nrf2 orchestrates gene expression that protects cells from oxidative damage and detoxifies xenobiotics; on the other hand, the same effects confer chemoresistance to cancer cells. It is important to discern when and how to manipulate Nrf2 and so we can make use of its advantages while minimizing potential disadvantages. The major negative sides of Nrf2 activation include promoting bioactivation of xenobiotics whose glucuronide conjugation form is genotoxic and forms adducts with DNA [186–188], neutralizing the chemotherapeutic effects in which oxidative stress plays a significant role in mediating cytotoxicity to cancer cells, and facilitating drug excretion from cell through increasing the expression levels of multidrug resistant pumps. Thereby, to minimize potential disadvantages, the use of phytoagents as Nrf2 activators for chemoprevention should carefully avoid coadministration of drugs that are bioactivated by Nrf2-regulated phase II enzyme processing. On the other hand, for pro-oxidant cancer chemotherapy, Nrf2 activation is deemed as a side-effect and should be suppressed by coadministration of Nrf2 inhibitor [185]. Still, more future studies are required to confirm these points and thus provide a more accurate prediction, and therefore application, of phytoagents in cancer management.

For phytochemicals that function as both antioxidants and prooxidants, further characterization of the factors that determine the transition from antioxidative to prooxidative effects in the biosystem is essential. One contributing factor is the presence of transition metals. In addition, the doses of phytochemicals used in each treatment at different times may be crucial. In this regard, we propose some considerations on context-dependent, dual function of phytoagents that may help to understand and to predict the chemotherapeutic role of phytoagents. By comparing normal and cancer-bearing individuals, we know that the oxidative DNA marker 8-oxoG correlates well with basal redox level [8, 189]. Cancer cells with higher basal redox level demonstrated elevated levels of 8-oxoG, whereas normal cells had lower levels of basal redox level and 8-oxoG. The overexpression of transferrin receptor in cancer cells increased intracellular level of ferrous ions and, presumably, increased the rate of the Fenton reaction. It can be assumed that high levels of ferrous ions in cancer cells switch the functions of phyto-antioxidants to those of pro-oxidants resulting in further elevation of ROS level in cancer cells but not in normal cells, and the selective killing of cancer cells. More studies are required to determine the concentration threshold of metal ions that switche phytoagents to their prooxidant roles, so that potential chemotherapeutic applications can be better characterized. In summary, two main points form the base of the concept of the context-dependent dual role of phytoagents. One is the level of intracellular level of transition metal ions and the other is the basal redox level. The higher the two, the more likely the agent to produce a pro-oxidant effect, whereas the lower the two, the more likely the agent to produce an antioxidant effect.

Continued rigorous research to identify molecular targets and conduct human studies with bioactive phytochemicals are important to provide potential alternatives or novel approaches for plant-based cancer prevention or therapy. It is likely that the anticancer properties of phytochemicals result from modulation of a number of molecular mechanisms that regulate different stages of carcinogenesis. In this regard, increased antioxidant strength may be important prior to dysregulation of signaling pathways during tumorigenesis, whereas prooxidant cytotoxicity may be critical in eliminating transformed tumor cells that exhibit dysregulated redox balance and metal ion absorption. In conclusion, careful dose-response and stage-dependent studies that compare enhancement of antioxidant capacity and induction of oxidative stress by phytochemicals are essential to clarify when and to what extent these phytoagents can be used in cancer prevention or therapy. 

## References

[1] Wolters S, Schumacher B (2013). Genome maintenance and transcription integrity in aging and disease. *Frontiers in Genetics*.

[2] Yin H, Xu L, Porter NA (2011). Free radical lipid peroxidation: mechanisms and analysis. *Chemical Reviews*.

[3] Grimm S, Höhn A, Grune T (2012). Oxidative protein damage and the proteasome. *Amino Acids*.

[4] Dizdaroglu M, Jaruga P, Birincioglu M, Rodriguez H (2002). Free radical-induced damage to DNA: mechanisms and measurement. *Free Radical Biology and Medicine*.

[5] Maynard S, Schurman SH, Harboe C, de Souza-Pinto NC, Bohr VA (2009). Base excision repair of oxidative DNA damage and association with cancer and aging. *Carcinogenesis*.

[6] Shaheen M, Shanmugam I, Hromas R (2010). The role of PCNA posttranslational modifications in translesion synthesis. *Journal of Nucleic Acids*.

[7] Jones S, Chen W-D, Parmigiani G (2008). Comparative lesion sequencing provides insights into tumor evolution. *Proceedings of the National Academy of Sciences of the United States of America*.

[8] Valavanidis A, Vlachogianni T, Fiotakis C (2009). 8-hydroxy-2′-deoxyguanosine (8-OHdG): a critical biomarker of oxidative stress and carcinogenesis. *Journal of Environmental Science and Health: Part C*.

[9] Lagadu S, Lechevrel M, Sichel F (2010). 8-oxo-7,8-dihydro-2′-deoxyguanosine as a biomarker of oxidative damage in oesophageal cancer patients: lack of association with antioxidant vitamins and polymorphism of hOGG1 and GST. *Journal of Experimental and Clinical Cancer Research*.

[10] Bartsch H, Nair J (2004). Oxidative stress and lipid peroxidation-derived DNA-lesions in inflammation driven carcinogenesis. *Cancer Detection and Prevention*.

[11] Wang M, Dhingra K, Hittelman WN, Liehr JG, De Andrade M, Li D (1996). Lipid peroxidation-induced putative malondialdehyde-DNA adducts in human breast tissues. *Cancer Epidemiology Biomarkers and Prevention*.

[12] Kaur S, Greaves P, Cooke DN (2007). Breast cancer prevention by green tea catechins and black tea theaflavins in the C3(1) SV40 T,t antigen transgenic mouse model is accompanied by increased apoptosis and a decrease in oxidative DNA adducts. *Journal of Agricultural and Food Chemistry*.

[13] Machowetz A, Poulsen HE, Gruendel S (2007). Effect of olive oils on biomarkers of oxidative DNA stress in Northern and Southern Europeans. *The FASEB Journal*.

[14] Birben E, Sahiner UM, Sackesen C, Erzurum S, Kalayci O (2012). Oxidative stress and antioxidant defense. *World Allergy Organization Journal*.

[15] Surh Y-J (2003). Cancer chemoprevention with dietary phytochemicals. *Nature Reviews Cancer*.

[16] Antonenkov VD, Grunau S, Ohlmeier S, Hiltunen JK (2010). Peroxisomes are oxidative organelles. *Antioxidants and Redox Signaling*.

[17] Sun X, Ai M, Wang Y (2013). Selective induction of tumor cell apoptosis by a novel P450-mediated reactive oxygen species (ROS) inducer methyl 3-(4-nitrophenyl) propiolate. *Journal of Biological Chemistry*.

[18] Zanotto-Filho A, Schroder R, Moreira JCF (2008). Xanthine oxidase-dependent ROS production mediates vitamin A pro-oxidant effects in cultured Sertoli cells. *Free Radical Research*.

[19] Taibi G, Carruba G, Miceli V, Cocciadiferro L, Cucchiara A, Nicotra CMA (2010). Sildenafil protects epithelial cell through the inhibition of xanthine oxidase and the impairment of ROS production. *Free Radical Research*.

[20] Beak SM, Lee YS, Kim J-A (2004). NADPH oxidase and cyclooxygenase mediate the ultraviolet B-induced generation of reactive oxygen species and activation of nuclear factor-*κ*B in HaCaT human keratinocytes. *Biochimie*.

[21] Matthias C, Schuster MT, Zieger S, Harreus U (2006). COX-2 inhibitors celecoxib and rofecoxib prevent oxidative DNA fragmentation. *Anticancer Research*.

[22] Los M, Schenk H, Hexel K, Baeuerle PA, Droge W, Schulze-Osthoff K (1995). IL-2 gene expression and NF-*κ* B activation through CD28 requires reactive oxygen production by 5-lipoxygenase. *EMBO Journal*.

[23] Edderkaoui M, Hong P, Vaquero EC (2005). Extracellular matrix stimulates reactive oxygen species production and increases pancreatic cancer cell survival through 5-lipoxygenase and NADPH oxidase. *American Journal of Physiology Gastrointestinal and Liver Physiology*.

[24] Grimm MJ, Vethanayagam RR, Almyroudis NG (2013). Monocyte- and macrophage-targeted NADPH oxidase mediates antifungal host defense and regulation of acute inflammation in mice. *Journal of Immunology*.

[25] Almyroudis NG, Grimm MJ, Davidson BA, Röhm M, Urban CF, Segal BH (2013). NETosis and NADPH oxidase: at the intersection of host defense, inflammation, and injury. *Frontiers in Immunology*.

[26] Nussbaum C, Klinke A, Adam M, Baldus S, Sperandio M (2013). Myeloperoxidase: a leukocyte-derived protagonist of inflammation and cardiovascular disease. *Antioxidants and Redox Signaling*.

[27] Tidén A-K, Sjögren T, Svensson M (2011). 2-thioxanthines are mechanism-based inactivators of myeloperoxidase that block oxidative stress during inflammation. *Journal of Biological Chemistry*.

[28] Trinchieri G (2012). Cancer and inflammation: an old intuition with rapidly evolving new concepts. *Annual Review of Immunology*.

[29] Stohs SJ, Bagchi D (1995). Oxidative mechanisms in the toxicity of metal ions. *Free Radical Biology and Medicine*.

[30] Lee JC, Son YO, Pratheeshkumar P, Shi X (2012). Oxidative stress and metal carcinogenesis. *Free Radical Biology and Medicine*.

[31] Sies H (1993). Strategies of antioxidant defense. *European Journal of Biochemistry*.

[32] Arosio P, Levi S (2002). Ferritin, iron homeostasis, and oxidative damage. *Free Radical Biology and Medicine*.

[33] Wu WS, Zhao YS, Shi ZH (2013). Mitochondrial ferritin attenuates *β*-amyloid-induced neurotoxicity: reduction in oxidative damage through the Erk/P38 mitogen-activated protein kinase pathways. *Antioxidants and Redox Signaling*.

[34] Luck AN, Mason AB (2012). Transferrin-mediated cellular iron delivery. *Current Topics Membranes*.

[35] Johannesson T, Kristinsson J, Torsdottir G, Snaedal J (2012). Ceruloplasmin (Cp) and iron in connection with Parkinson's disease (PD) and Alzheimer's disease (AD). *Laeknabladid*.

[36] Qu W, Pi J, Waalkes MP (2013). Metallothionein blocks oxidative DNA damage in vitro. *Archives of Toxicology*.

[37] Beutler E (2008). Glucose-6-phosphate dehydrogenase deficiency: a historical perspective. *Blood*.

[38] Kobayashi M, Yamamoto M (2005). Molecular mechanisms activating the Nrf2-Keap1 pathway of antioxidant gene regulation. *Antioxidants and Redox Signaling*.

[39] Niture SK, Khatri R, Jaiswal AK (2013). Regulation of Nrf2—an update. *Free
Radical Biology and Medicine*.

[40] Kensler TW, Wakabayashi N, Biswal S (2007). Cell survival responses to environmental stresses via the Keap1-Nrf2-ARE pathway. *Annual Review of Pharmacology and Toxicology*.

[41] Kim KC, Kang KA, Zhang R (2010). Up-regulation of Nrf2-mediated heme oxygenase-1 expression by eckol, a phlorotannin compound, through activation of Erk and PI3K/Akt. *International Journal of Biochemistry and Cell Biology*.

[42] Kaspar JW, Niture SK, Jaiswal AK (2009). Nrf2:INrf2 (Keap1) signaling in oxidative stress. *Free Radical Biology and Medicine*.

[43] Kim J, Cha Y-N, Surh Y-J (2010). A protective role of nuclear factor-erythroid 2-related factor-2 (Nrf2) in inflammatory disorders. *Mutation Research*.

[44] Kundu JK, Surh Y-J (2010). Nrf2-keap1 signaling as a potential target for chemoprevention of inflammation-associated carcinogenesis. *Pharmaceutical Research*.

[45] Na H-K, Kim E-H, Jung J-H, Lee H-H, Hyun J-W, Surh Y-J (2008). (−)-Epigallocatechin gallate induces Nrf2-mediated antioxidant enzyme expression via activation of PI3K and ERK in human mammary epithelial cells. *Archives of Biochemistry and Biophysics*.

[46] Knobel PA, Marti TM (2011). Translesion DNA synthesis in the context of cancer research. *Cancer Cell International*.

[47] Sale JE (2013). Translesion DNA synthesis and mutagenesis in eukaryotes. *Cold Spring Harbor Perspectives in Biology*.

[48] Benderoth M, Textor S, Windsor AJ, Mitchell-Olds T, Gershenzon J, Kroymann J (2006). Positive selection driving diversification in plant secondary metabolism. *Proceedings of the National Academy of Sciences of the United States of America*.

[49] Newman DJ, Cragg GM (2007). Natural products as sources of new drugs over the last 25 years. *Journal of Natural Products*.

[50] Pan L, Chai H, Kinghorn AD (2010). The continuing search for antitumor agents from higher plants. *Phytochemistry Letters*.

[51] Singh S (2007). From exotic spice to modern drug?. *Cell*.

[52] Harvey AL (2008). Natural products in drug discovery. *Drug Discovery Today*.

[53] Li JW-H, Vederas JC (2009). Drug discovery and natural products: end of an era or an endless frontier?. *Science*.

[54] Lee W-L, Shiau J-Y, Shyur L-F (2012). Taxol, camptothecin and beyond for cancer therapy. *Advances in Botanical Research*.

[55] Hsan KM, Chen C-C, Shyur L-F (2010). Current research and development of chemotherapeutic agents for melanoma. *Cancers*.

[56] Antoslewicz J, Ziolkowski W, Kar S, Powolny AA, Singh SV (2008). Role of reactive oxygen intermediates in cellular responses to dietary cancer chemopreventive agents. *Planta Medica*.

[57] Neves AR, Lucio M, Lima JLC, Reis S (2012). Resveratrol in medicinal chemistry: a critical review of its pharmacokinetics, drug-delivery, and membrane interactions. *Current Medicinal Chemistry*.

[58] Li H-Q, Luo Y, Qiao C-H (2012). The mechanisms of anticancer agents by genistein and synthetic derivatives of isoflavone. *Mini-Reviews in Medicinal Chemistry*.

[59] López-Lázaro M (2008). Anticancer and carcinogenic properties of curcumin: considerations for its clinical development as a cancer chemopreventive and chemotherapeutic agent. *Molecular Nutrition and Food Research*.

[60] Lambert JD, Elias RJ (2010). The antioxidant and pro-oxidant activities of green tea polyphenols: a role in cancer prevention. *Archives of Biochemistry and Biophysics*.

[61] Lee W-L, Wen T-N, Shiau J-Y, Shyur L-F (2010). Differential proteomic profiling identifies novel molecular targets of paclitaxel and phytoagent deoxyelephantopin against mammary adenocarcinoma cells. *Journal of Proteome Research*.

[62] Huang C-C, Lo C-P, Chiu C-Y, Shyur L-F (2010). Deoxyelephantopin, a novel multifunctional agent, suppresses mammary tumour growth and lung metastasis and doubles survival time in mice. *British Journal of Pharmacology*.

[63] Lee W-L, Shyur L-F (2012). Deoxyelephantopin impedes mammary adenocarcinoma cell motility by inhibiting calpain-mediated adhesion dynamics and inducing reactive oxygen species and aggresome formation. *Free Radical Biology and Medicine*.

[64] Efferth T (2006). Molecular pharmacology and pharmacogenomics of artemisinin and its derivatives in cancer cells. *Current Drug Targets*.

[65] Kim SL, Trang KT, Kim SH (2012). Parthenolide suppresses tumor growth in a xenograft model of colorectal cancer cells by inducing mitochondrial dysfunction and apoptosis. *International Journal of Oncology*.

[66] Oka D, Nishimura K, Shiba M (2007). Sesquiterpene lactone parthenolide suppresses tumor growth in a xenograft model of renal cell carcinoma by inhibiting the activation of NF-*κ*B. *International Journal of Cancer*.

[67] Sweeney CJ, Mehrotra S, Sadaria MR (2005). The sesquiterpene lactone parthenolide in combination with docetaxel reduces metastasis and improves survival in a xenograft model of breast cancer. *Molecular Cancer Therapeutics*.

[68] Lee KW, Bode AM, Dong Z (2011). Molecular targets of phytochemicals for cancer prevention. *Nature Reviews Cancer*.

[69] Mandel SA, Amit T, Kalfon L, Reznichenko L, Weinreb O, Youdim MB (2008). Cell signaling pathways and iron chelation in the neurorestorative activity of green tea polyphenols: special reference to epigallocatechin gallate (EGCG). *Journal of Alzheimer's Disease*.

[70] Hider RC, Liu ZD, Khodr HH (2001). Metal chelation of polyphenols. *Methods in Enzymology*.

[71] Hayes JD, McMahon M, Chowdhry S, Dinkova-Kostova AT (2010). Cancer chemoprevention mechanisms mediated through the keap1-Nrf2 pathway. *Antioxidants and Redox Signaling*.

[72] Gerhäuser C, Klimo K, Heiss E (2003). Mechanism-based in vitro screening of potential cancer chemopreventive agents. *Mutation Research*.

[73] Papa S, Bubici C, Pham CG, Zazzeroni F, Franzoso G (2005). NF-*κ*B meets ROS: an “iron-ic” encounter. *Cell Death and Differentiation*.

[74] Meng Z, Yan C, Deng Q, Gao DF, Niu XL (2013). Curcumin inhibits LPS-induced inflammation in rat vascular smooth muscle cells in vitro via ROS-relative TLR4-MAPK/NF-*κ*B pathways. *Acta Pharmacologica Sinica*.

[75] Qi S, Xin Y, Guo Y (2012). Ampelopsin reduces endotoxic inflammation via repressing ROS-mediated activation of PI3K/Akt/NF-*κ*B signaling pathways. *International Immunopharmacology*.

[76] Ren D, Wang H, Liu J, Zhang M, Zhang W (2012). ROS-induced ZNF580 expression: a key role for H_2_O_2_NF-*κ*B signaling pathway in vascular endothelial inflammation. *Molecular and Cellular Biochemistry*.

[77] Yang C, Yang Z, Zhang M (2011). Hydrogen sulfide protects against chemical hypoxia-induced cytotoxicity and inflammation in hacat cells through inhibition of ROS/NF-*κ*B/COX-2 pathway. *PLoS One*.

[78] Box HC, Patrzyc HB, Budzinski EE (2012). Profiling oxidative DNA damage: effects of antioxidants. *Cancer Science*.

[79] Farias MS, Budni P, Ribeiro CM, Parisotto EB, Santos CE, Dias JF (2012). Antioxidant supplementation attenuates oxidative stress in chronic hepatitis C patients. *Gastroenterología y Hepatología*.

[80] Singh N, Bhardwaj P, Pandey RM, Saraya A (2012). Oxidative stress and antioxidant capacity in patients with chronic pancreatitis with and without diabetes mellitus. *Indian Journal of Gastroenterology*.

[81] Puertollano MA, Puertollano E, De Cienfuegos GA, De Pablo MA (2011). Dietary antioxidants: immunity and host defense. *Current Topics in Medicinal Chemistry*.

[82] McCall MR, Frei B (1999). Can antioxidant vitamins materially reduce oxidative damage in humans?. *Free Radical Biology and Medicine*.

[83] Myung S-K, Kim Y, Ju W, Choi HJ, Bae WK (2010). Effects of antioxidant supplements on cancer prevention: meta-analysis of randomized controlled trials. *Annals of Oncology*.

[84] Chang YJ, Myung S-K, Chung ST (2011). Effects of vitamin treatment or supplements with purported antioxidant properties on skin cancer prevention: a meta-analysis of randomized controlled trials. *Dermatology*.

[85] Mosby TT, Cosgrove M, Sarkardei S, Platt KL, Kaina B (2012). Nutrition in adult and childhood cancer: role of carcinogens and anti-carcinogens. *Anticancer Research*.

[86] Cheung FS, Lovicu FJ, Reichardt JK (2012). Current progress in using vitamin D and its analogs for cancer prevention and treatment. *Expert Review of Anticancer Therapy*.

[87] Garland CF, French CB, Baggerly LL, Heaney RP (2011). Vitamin D supplement doses and serum 25-Hydroxyvitamin D in the range associated with cancer prevention. *Anticancer Research*.

[88] Gaziano JM, Sesso HD, Christen WG (2012). Multivitamins in the prevention of cancer in men: the Physicians' Health Study II randomized controlled trial. *The Journal of the American Medical Association*.

[89] Sram RJ, Farmer P, Singh R (2009). Effect of vitamin levels on biomarkers of exposure and oxidative damage—the EXPAH study. *Mutation Research*.

[90] Yan Y, Yang J-Y, Mou Y-H, Wang L-H, Zhou Y-N, Wu C-F (2012). Differences in the activities of resveratrol and ascorbic acid in protection of ethanol-induced oxidative DNA damage in human peripheral lymphocytes. *Food and Chemical Toxicology*.

[91] Collins BH, Horská A, Hotten PM, Riddoch C, Collins AR (2001). Kiwifruit protects against oxidative DNA damage in human cells and in vitro. *Nutrition and Cancer*.

[92] Fiorentino A, D'abrosca B, Pacifico S, Mastellone C, Scognamiglio M, Monaco P (2009). Identification and assessment of antioxidant capacity of phytochemicals from kiwi fruits. *Journal of Agricultural and Food Chemistry*.

[93] Kapiszewska M, Soltys E, Visioli F, Cierniak A, Zajac G (2005). The protective ability of the Mediterranean plant extracts against the oxidative DNA damage. The role of the radical oxygen species and the polyphenol content. *Journal of Physiology and Pharmacology*.

[94] Viladomiu M, Hontecillas R, Lu P, Bassaganya-Riera J (2013). Preventive and prophylactic mechanisms of action of pomegranate bioactive constituents. *Evidence-Based Complementary and Alternative Medicine*.

[95] Jurenka J (2008). Therapeutic applications of pomegranate (*Punica granatum* L.): a review. *Alternative Medicine Review*.

[96] Bishayee A, Bhatia D, Thoppil RJ, Darvesh AS, Nevo E, Lansky EP (2011). Pomegranate-mediated chemoprevention of experimental hepatocarcinogenesis involves Nrf2-regulated antioxidant mechanisms. *Carcinogenesis*.

[97] Thoppil RJ, Bhatia D, Barnes KF (2012). Black currant anthocyanins abrogate oxidative stress through Nrf2- mediated antioxidant mechanisms in a rat model of hepatocellular carcinoma. *Currant Cancer Drug Targets*.

[98] Romagnolo DF, Selmin OI (2012). Flavonoids and cancer prevention: a review of the evidence. *Journal of Nutrition in Gerontology and Geriatrics 
*.

[99] Hayes JD, McMahon M (2001). Molecular basis for the contribution of the antioxidant responsive element to cancer chemoprevention. *Cancer Letters*.

[100] Na H-K, Surh Y-J (2008). Modulation of Nrf2-mediated antioxidant and detoxifying enzyme induction by the green tea polyphenol EGCG. *Food and Chemical Toxicology*.

[101] Surh Y-J, Kundu JK, Na H-K (2008). Nrf2 as a master redox switch in turning on the cellular signaling involved in the induction of cytoprotective genes by some chemopreventive phytochemicals. *Planta Medica*.

[102] Platet N, Cathiard AM, Gleizes M, Garcia M (2004). Estrogens and their receptors in breast cancer progression: a dual role in cancer proliferation and invasion. *Critical Reviews in Oncology/Hematology*.

[103] Lee AJ, Cai MX, Thomas PE, Conney AH, Zhu BT (2003). Characterization of the oxidative metabolites of 17*β*-estradiol and estrone formed by 15 selectively expressed human cytochrome P450 isoforms. *Endocrinology*.

[104] Aiyer HS, Vadhanam MV, Stoyanova R, Caprio GD, Clapper ML, Gupta RC (2008). Dietary berries and ellagic acid prevent oxidative DNA damage and modulate expression of DNA repair genes. *International Journal of Molecular Sciences*.

[105] Aiyer HS, Kichambare S, Gupta RC (2008). Prevention of oxidative DNA damage by bioactive berry components. *Nutrition and Cancer*.

[106] Wondrak GT (2009). Redox-directed cancer therapeutics: molecular mechanisms and opportunities. *Antioxidants and Redox Signaling*.

[107] Yang J-C, Lu M-C, Lee C-L (2011). Selective targeting of breast cancer cells through ROS-mediated mechanisms potentiates the lethality of paclitaxel by a novel diterpene, gelomulide K. *Free Radical Biology and Medicine*.

[108] Trachootham D, Alexandre J, Huang P (2009). Targeting cancer cells by ROS-mediated mechanisms: a radical therapeutic approach?. *Nature Reviews Drug Discovery*.

[109] Bystrom LM, Guzman ML, Rivella S (2013). Iron and reactive oxygen species: friends or foes of cancer cells?. *Antioxidants and Redox Signaling*.

[110] Calzolari A, Oliviero I, Deaglio S (2007). Transferrin receptor 2 is frequently expressed in human cancer cell lines. *Blood Cells, Molecules, and Diseases*.

[111] Daniels TR, Bernabeu E, Rodríguez JA (2012). The transferrin receptor and the targeted delivery of therapeutic agents against cancer. *Biochimica et Biophysica Acta*.

[112] You BR, Kim SZ, Kim SH, Park WH (2011). Gallic acid-induced lung cancer cell death is accompanied by ROS increase and glutathione depletion. *Molecular and Cellular Biochemistry*.

[113] Chen G, Chen Z, Hu Y, Huang P (2011). Inhibition of mitochondrial respiration and rapid depletion of mitochondrial glutathione by *β*-phenethyl isothiocyanate: mechanisms for anti-leukemia activity. *Antioxidants and Redox Signaling*.

[114] Locatelli C, Leal PC, Yunes RA, Nunes RJ, Creczynski-Pasa TB (2009). Gallic acid ester derivatives induce apoptosis and cell adhesion inhibition in melanoma cells: the relationship between free radical generation, glutathione depletion and cell death. *Chemico-Biological Interactions*.

[115] Piwocka K, Jaruga E, Skierski J, Gradzka I, Sikora E (2001). Effect of glutathione depletion on caspase-3 independent apoptosis pathway induced by curcumin in Jurkat cells. *Free Radical Biology and Medicine*.

[116] Pandey MK, Kumar S, Thimmulappa RK, Parmar VS, Biswal S, Watterson AC (2011). Design, synthesis and evaluation of novel PEGylated curcumin analogs as potent Nrf2 activators in human bronchial epithelial cells. *European Journal of Pharmaceutical Sciences*.

[117] Yang C, Zhang X, Fan H, Liu Y (2009). Curcumin upregulates transcription factor Nrf2, HO-1 expression and protects rat brains against focal ischemia. *Brain Research*.

[118] Kang ES, Kim GH, Kim HJ (2008). Nrf2 regulates curcumin-induced aldose reductase expression indirectly via nuclear factor-*κ*B. *Pharmacological Research*.

[119] Hou D-X, Korenori Y, Tanigawa S (2011). Dynamics of Nrf2 and Keap1 in ARE-mediated NQO1 expression by wasabi 6-(methylsulfinyl)hexyl isothiocyanate. *Journal of Agricultural and Food Chemistry*.

[120] Wagner AE, Boesch-Saadatmandi C, Dose J, Schultheiss G, Rimbach G (2012). Anti-inflammatory potential of allyl-isothiocyanate—role of Nrf2, NF-*κ*B and microRNA-155. *Journal of Cellular and Molecular Medicine*.

[121] Ernst IM, Wagner AE, Schuemann C (2011). Allyl-, butyl- and phenylethyl-isothiocyanate activate Nrf2 in cultured fibroblasts. *Pharmacological Research*.

[122] Schumacker PT (2006). Reactive oxygen species in cancer cells: live by the sword, die by the sword. *Cancer Cell*.

[123] Warburg O (1956). On the origin of cancer cells. *Science*.

[124] Yoshii Y, Furukawa T, Yoshii H (2009). Cytosolic acetyl-CoA synthetase affected tumor cell survival under hypoxia: the possible function in tumor acetyl-CoA/acetate metabolism. *Cancer Science*.

[125] Simizu S, Takada M, Umezawa K, Imoto M (1998). Requirement of caspase-3(-like) protease-mediated hydrogen peroxide production for apoptosis induced by various anticancer drugs. *Journal of Biological Chemistry*.

[126] Fang J, Seki T, Maeda H (2009). Therapeutic strategies by modulating oxygen stress in cancer and inflammation. *Advanced Drug Delivery Reviews*.

[127] Raj L, Ide T, Gurkar AU (2011). Selective killing of cancer cells by a small molecule targeting the stress response to ROS. *Nature*.

[128] Trachootham D, Zhou Y, Zhang H (2006). Selective killing of oncogenically transformed cells through a ROS-mediated mechanism by *β*-phenylethyl isothiocyanate. *Cancer Cell*.

[129] Hail N, Cortes M, Drake EN, Spallholz JE (2008). Cancer chemoprevention: a radical perspective. *Free Radical Biology and Medicine*.

[130] Powolny AA, Singh SV (2008). Multitargeted prevention and therapy of cancer by diallyl trisulfide and related Allium vegetable-derived organosulfur compounds. *Cancer Letters*.

[131] Murata M, Yamashita N, Inoue S, Kawanishi S (2000). Mechanism of oxidative DNA damage induced by carcinogenic allyl isothiocyanate. *Free Radical Biology and Medicine*.

[132] Ahsan H, Hadi SM (1998). Strand scission in DNA induced by curcumin in the presence of Cu(II). *Cancer Letters*.

[133] Ghantous A, Gali-Muhtasib H, Vuorela H, Saliba NA, Darwiche N (2010). What made sesquiterpene lactones reach cancer clinical trials?. *Drug Discovery Today*.

[134] Singh NP, Verma KB (2002). Case report of a laryngeal squamous cell carcinoma treated with artesunate. *Archive of Oncology*.

[135] Curry EA, Murry DJ, Yoder C (2004). Phase I dose escalation trial of feverfew with standardized doses of parthenolide in patients with cancer. *Investigational New Drugs*.

[136] As ML Completed phase 2 clinical trials for parthenolide in treating allergic contact dermatitis. http://clinicaltrials.gov/ct2/show/NCT00133341?term=Parthenolide&rank=1.

[137] Singh NP, Panwar VK (2006). Case report of a pituitary macroadenoma treated with artemether. *Integrative Cancer Therapies*.

[138] Zhang Z-Y, Yu S-Q, Miao L-Y (2008). Artesunate combined with vinorelbine plus cisplatin in treatment of advanced non-small cell lung cancer: a randomized controlled trial. *Zhong Xi Yi Jie He Xue Bao*.

[139] Efferth T (2007). Willmar Schwabe Award 2006: antiplasmodial and antitumor activity of artemisinin—from bench to bedside. *Planta Medica*.

[140] Gill KK, Kaddoumi A, Nazzal S (2012). Mixed micelles of PEG2000-DSPE and vitamin-E TPGS for concurrent delivery of paclitaxel and parthenolide: enhanced chemosenstization and antitumor efficacy against non-small cell lung cancer (NSCLC) cell lines. *European Journal of Pharmaceutical Sciences*.

[141] Sohma I, Fujiwara Y, Sugita Y (2011). Parthenolide, an NF-*κ*B inhibitor, suppresses tumor growth and enhances response to chemotherapy in gastric cancer. *Cancer Genomics and Proteomics*.

[142] Kreuger MR, Grootjans S, Biavatti MW, Vandenabeele P, D'herde K (2012). Sesquiterpene lactones as drugs with multiple targets in cancer treatment: focus on parthenolide. *Anti-Cancer Drugs*.

[143] Ponka P, Beaumont C, Richardson DR (1998). Function and regulation of transferrin and ferritin. *Seminars in Hematology*.

[144] Harris ED (1992). Regulation of antioxidant enzymes. *The FASEB Journal*.

[145] Gupte A, Mumper RJ (2009). Elevated copper and oxidative stress in cancer cells as a target for cancer treatment. *Cancer Treatment Reviews*.

[146] Coates RJ, Weiss NS, Daling JR, Rettmer RL, Warnick GR (1989). Cancer risk in relation to serum copper levels. *Cancer Research*.

[147] Kwok JC, Richardson DR (2002). The iron metabolism of neoplastic cells: alterations that facilitate proliferation?. *Critical Reviews in Oncology/Hematology*.

[148] Wu T, Sempos CT, Freudenheim JL, Muti P, Smit E (2004). Serum iron, copper and zinc concentrations and risk of cancer mortality in US adults. *Annals of Epidemiology*.

[149] Kuo HW, Chen SF, Wu CC, Chen DR, Lee JH (2002). Serum and tissue trace elements in patients with breast cancer in Taiwan. *Biological Trace Element Research*.

[150] Chan A, Wong F, Arumanayagam M (1993). Serum ultrafiltrable copper, total copper and caeruloplasmin concentrations in gynaecological carcinomas. *Annals of Clinical Biochemistry*.

[151] Diez M, Arroyo M, Cerdan FJ, Munoz M, Martin MA, Balibrea JL (1989). Serum and tissue trace metal levels in lung cancer. *Oncology*.

[152] Habib FK, Dembinski TC, Stitch SR (1980). The zinc and copper content of blood leucocytes and plasma from patients with benign and malignant prostates. *Clinica Chimica Acta*.

[153] Mazdak H, Yazdekhasti F, Movahedian A, Mirkheshti N, Shafieian M (2010). The comparative study of serum iron, copper, and zinc levels between bladder cancer patients and a control group. *International Urology and Nephrology*.

[154] Scanni A, Licciardello L, Trovato M, Tomirotti M, Biraghi M (1977). Serum copper and ceruloplasmin levels in patients with neoplasias localized in the stomach, large intestine or lung. *Tumori*.

[155] Zuo XL, Chen JM, Zhou X, Li XZ, Mei GY (2006). Levels of selenium, zinc, copper, and antioxidant enzyme activity in patients with leukemia. *Biological Trace Element Research*.

[156] Silva MP, Soave DF, Ribeiro-Silva A, Poletti ME (2012). Trace elements as tumor biomarkers and prognostic factors in breast cancer: a study through energy dispersive x-ray fluorescence. *BMC Research Notes*.

[157] Bryan SE, Vizard DL, Beary DA, Labiche RA, Hardy KJ (1981). Partitioning of zinc and copper within subnuclear nucleoprotein particles. *Nucleic Acids Research*.

[158] Prousek J (2007). Fenton chemistry in biology and medicine. *Pure and Applied Chemistry*.

[159] Jeong JB, Seo EW, Jeong HJ (2009). Effect of extracts from pine needle against oxidative DNA damage and apoptosis induced by hydroxyl radical via antioxidant activity. *Food and Chemical Toxicology*.

[160] Jeong JB, De Lumen BO, Jeong HJ (2010). Lunasin peptide purified from Solanum nigrum L. protects DNA from oxidative damage by suppressing the generation of hydroxyl radical via blocking fenton reaction. *Cancer Letters*.

[161] Khokhar S, Apenten RKO (2003). Iron binding characteristics of phenolic compounds: some tentative structure-activity relations. *Food Chemistry*.

[162] Andjelković M, Camp JV, Meulenaer BD (2006). Iron-chelation properties of phenolic acids bearing catechol and galloyl groups. *Food Chemistry*.

[163] Ba Q, Zhou N, Duan J (2012). Dihydroartemisinin exerts its anticancer activity through depleting cellular iron via transferrin receptor-1. *PLoS One*.

[164] Merlot AM, Kalinowski DS, Richardson DR (2013). Novel chelators for cancer treatment: where are we now?. *Antioxid Redox Signal*.

[165] Markova NG, Karaman-Jurukovska N, Dong KK, Damaghi N, Smiles KA, Yarosh DB (2009). Skin cells and tissue are capable of using l-ergothioneine as an integral component of their antioxidant defense system. *Free Radical Biology and Medicine*.

[166] Zhu B-Z, Mao L, Fan R-M (2011). Ergothioneine prevents copper-induced oxidative damage to DNA and protein by forming a redox-inactive ergothioneine-copper complex. *Chemical Research in Toxicology*.

[167] Halliwell B (1999). Antioxidant defence mechanisms: from the beginning to the end (of the beginning). *Free Radical Research*.

[168] Inoue S, Ito K, Yamamoto K, Kawanishi S (1992). Caffeic acid causes metal-dependent damage to cellular and isolated DNA through H_2_O_2_ formation. *Carcinogenesis*.

[169] Yamashita N, Tanemura H, Kawanishi S (1999). Mechanism of oxidative DNA damage induced by quercetin in the presence of Cu(II). *Mutation Research*.

[170] Bobrowska B, Skrajnowska D, Tokarz A (2011). Effect of Cu supplementation on genomic instability in chemically-induced mammary carcinogenesis in the rat. *Journal of Biomedical Science*.

[171] Bobrowska-Korczak B, Skrajnowska D, Tokarz A (2012). The effect of dietary zinc—and polyphenols intake on DMBA-induced mammary tumorigenesis in rats. *Journal of Biomedical Science*.

[172] Zheng L-F, Wei Q-Y, Cai Y-J (2006). DNA damage induced by resveratrol and its synthetic analogues in the presence of Cu (II) ions: mechanism and structure-activity relationship. *Free Radical Biology and Medicine*.

[173] Halliwell B (1996). Vitamin C: antioxidant or pro-oxidant in vivo?. *Free Radical Research*.

[174] Rehman A, Collis CS, Yang M (1998). The effects of iron and vitamin C co-supplementation on oxidative damage to DNA in healthy volunteers. *Biochemical and Biophysical Research Communications*.

[175] Torshina NR, Zhang JZ, Heck DE (2007). Catalytic therapy of cancer with porphyrins and ascorbate. *Cancer Letters*.

[176] Torshina NR, Zhang JZ, Heck DE (2010). Catalytic therapy of cancer with ascorbate and extracts of medicinal herbs. *Evidence-Based Complementary and Alternative Medicine*.

[177] Azmi AS, Bhat SH, Hanif S, Hadi SM (2006). Plant polyphenols mobilize endogenous copper in human peripheral lymphocytes leading to oxidative DNA breakage: a putative mechanism for anticancer properties. *The FEBS Letters*.

[178] Hadi SM, Ullah MF, Shamim U, Bhatt SH, Azmi AS (2010). Catalytic therapy of cancer by ascorbic acid involves redox cycling of exogenous/endogenous copper ions and generation of reactive oxygen species. *Chemotherapy*.

[179] Khan HY, Zubair H, Ullah MF, Ahmad A, Hadi SM (2011). Oral administration of copper to rats leads to increased lymphocyte cellular DNA degradation by dietary polyphenols: Implications for a cancer preventive mechanism. *BioMetals*.

[180] Zubair H, Khan HY, Ullah MF, Ahmad A, Wu D, Hadi SM (2012). Apogossypolone, derivative of gossypol, mobilizes endogenous copper in human peripheral lymphocytes leading to oxidative DNA breakage. *European Journal of Pharmaceutical Sciences*.

[181] Preston TJ, Henderson JT, McCallum GP, Wells PG (2009). Base excision repair of reactive oxygen species-initiated 7,8-dihydro-8-oxo-2′-deoxyguanosine inhibits the cytotoxicity of platinum anticancer drugs. *Molecular Cancer Therapeutics*.

[182] Das GC, Bacsi A, Shrivastav M, Hazra TK, Boldogh I (2006). Enhanced gamma-glutamylcysteine synthetase activity decreases drug-induced oxidative stress levels and cytotoxicity. *Molecular Carcinogenesis*.

[183] Glorieux C, Dejeans N, Sid B, Beck R, Calderon PB, Verrax J (2011). Catalase overexpression in mammary cancer cells leads to a less aggressive phenotype and an altered response to chemotherapy. *Biochemical Pharmacology*.

[184] Lau A, Villeneuve NF, Sun Z, Wong PK, Zhang DD (2008). Dual roles of Nrf2 in cancer. *Pharmacological Research*.

[185] Sporn MB, Liby KT (2012). NRF2 and cancer: the good, the bad and the importance of context. *Nature Reviews Cancer*.

[186] Ghaoui R, Sallustio BC, Burcham PC, Fontaine FR (2003). UDP-glucuronosyltransferase-dependent bioactivation of clofibric acid to a DNA-damaging intermediate in mouse hepatocytes. *Chemico-Biological Interactions*.

[187] Sallustio BC, Fishbein JC (2008). Glucuronidation-dependent toxicity and bioactivation. *Advances in Molecular Toxicology*.

[188] Sallustio BC, Harkin LA, Mann MC, Krivickas SJ, Burcham PC (1997). Genotoxicity of acyl glucuronide metabolites formed from clofibric acid and gemfibrozil: a novel role for phase-II-mediated bioactivation in the hepatocarcinogenicity of the parent aglycones?. *Toxicology and Applied Pharmacology*.

[189] Peddireddy V, Siva Prasad B, Gundimeda SD, Penagaluru PR, Mundluru HP (2012). Assessment of 8-oxo-7, 8-dihydro-2′-deoxyguanosine and malondialdehyde levels as oxidative stress markers and antioxidant status in non-small cell lung cancer. *Biomarkers*.

